# Advanced Cancer Liquid Biopsy Platform for miRNA Detection in Extracellular Vesicles Using CRISPR/Cas13a and Gold Nanoarrays

**DOI:** 10.1021/acsnano.5c06940

**Published:** 2025-07-28

**Authors:** Meizi Chen, Hye Kyu Choi, Li Ling Goldston, Yannan Hou, Caiping Jiang, Ki-Bum Lee

**Affiliations:** 1 Department of Chemistry and Chemical Biology, Rutgers, The State University of New Jersey,123 Bevier Road, Piscataway, New Jersey 08854, United States; 2 Department of Chemical and Biomolecular Engineering, Sogang University, Seoul 04107, Republic of Korea

**Keywords:** nano liquid biopsy, extracellular vesicle microRNA detection, aptamer-functionalized nanoarrays, CRISPR/Cas13a diagnostics, nanoscale reactors, vascularized tumor spheroid, noninvasive cancer detection

## Abstract

Liquid biopsy is a transformative, noninvasive tool for cancer diagnosis and monitoring, with the potential to revolutionize personalized medicine. In this study, we introduce an advanced liquid biopsy platform for highly sensitive and selective detection of extracellular vesicle (EV) microRNAs (miRNA-21 and miRNA-23a) as biomarkers for colorectal cancer. The platform combines two innovations: (1) gold nanoarrays with epithelial cell adhesion molecule (EpCAM)-specific aptamers to selectively isolate tumor-derived EVs from plasma and (2) CRISPR/Cas13a-encapsulated liposomes that fuse with EVs to form nanoscale reactors. Upon fusion, the CRISPR/Cas13a complex is activated by target miRNA, triggering trans-cleavage of RNA reporters and generating an amplified fluorescence signal for enhanced detection sensitivity. The assay achieves a linear detection range of 10 to 10^6^ EVs/μL and a detection limit of 2.5 × 10^1^ EVs/μL on the gold nanoarray. Its performance relies on three strategies: (i) EpCAM-mediated tumor EV enrichment, (ii) CRISPR/Cas13a-based collateral activity for ultrasensitive miRNA detection, and (iii) fluorescence signal enhancement via localized nanoreactors. Validation with a 2D SW480 cell model, a 3D vascularized tumor spheroid (VTS) model, and clinical plasma samples confirms diagnostic accuracy, with miRNA quantification comparable to RT-qPCR but without the need for labor-intensive RNA extraction and amplification. By integrating nanotechnology with CRISPR-based diagnostics, this platform bridges research and clinical translation, improving diagnostic precision and streamlining workflows. Future development will focus on multiplexed biomarker detection and single-EV analysis to reveal insights into EV heterogeneity and function in cancer. This technology supports the application in precision oncology, offering a tool for early detection, treatment monitoring, and therapeutic decision-making.

## Graphical abstract

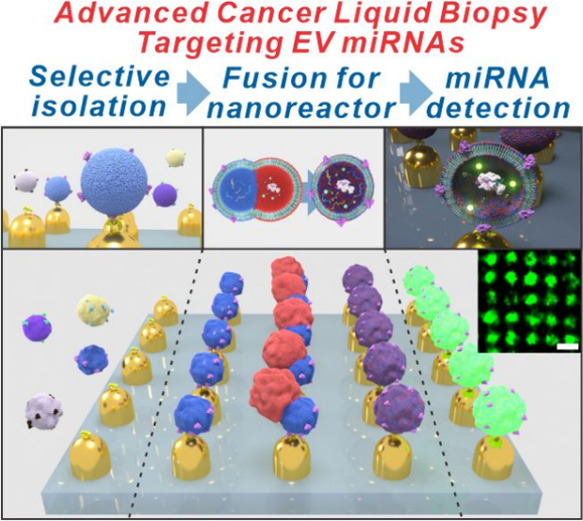

## Introduction

The integration of extracellular vesicle (EV)-based nanobiotechnology into early and comprehensive cancer diagnosis represents a transformative approach with immense potential to advance personalized medicine. EVs are nanoscale vesicles naturally secreted by cells, playing a crucial role in mediating intercellular communication.^1,2^ These vesicles are enriched with a diverse range of molecular cargo, including proteins, RNA, DNA, and lipids, which represent the physiological and pathological conditions of their parent cells.^3–8^ The ability of EVs to encapsulate such molecular signatures offers a unique window into the underlying biological mechanisms driving cancer development and progression.^9,10^ Furthermore, EVs possess distinctive properties, such as their capacity to circulate in various bodily fluids and to transport bioactive molecular contents, making them highly advantageous for clinical applications.^11,12^ These unique features position EVs as a robust platform for the early detection of cancer, enabling more precise and timely identification of the disease. Additionally, their molecular signatures hold the potential to guide personalized therapeutic strategies, aligning treatment approaches with the specific biological characteristics of an individual’s cancer.^13–15^

One of the noninvasive approaches for cancer diagnosis and monitoring is liquid biopsy. This technique involves collecting and analyzing nonsolid biofluids, such as blood, urine, or cerebrospinal fluid, to gain critical insights into the molecular characteristics of tumors.^16,17^ By detecting specific biomarkers, such as circulating tumor DNA (ctDNA), EVs, and circulating tumor cells (CTCs) within these fluids, liquid biopsy provides a comprehensive overview of the tumor’s genetic and molecular profile.^18–25^ This method holds great potential for real-time monitoring of disease progression, therapeutic response, and early detection of cancer recurrence, making it an invaluable tool in personalized oncology. Among the emerging biomarkers for cancer diagnostics, small EVs with size smaller than 200 nm in diameter have demonstrated remarkable potential due to their abundance and stability in the circulatory system^26–28^ [Figure 1A]. These attributes enable EVs to represent the physiological and pathological conditions of their parent cells. Consequently, EVs can serve as excellent biomarkers for noninvasive liquid biopsy, offering insights into tumor biology and facilitating early disease detection and monitoring. Within the cargo of EVs, microRNAs (miRNAs), a class of small noncoding RNAs, have been established as highly valuable diagnostic and prognostic biomarkers due to their pivotal roles in regulating gene expression.^29–31^ Encapsulated within the protective lipid bilayer of EVs, miRNAs are protected from enzymatic degradation by RNases, conferring their greater stability compared to cell-free miRNAs.^32,33^ This inherent stability enhances the reliability of EV miRNAs as biomarkers in liquid biopsy applications, providing a robust framework for cancer analysis and monitoring.

**Figure 1:**
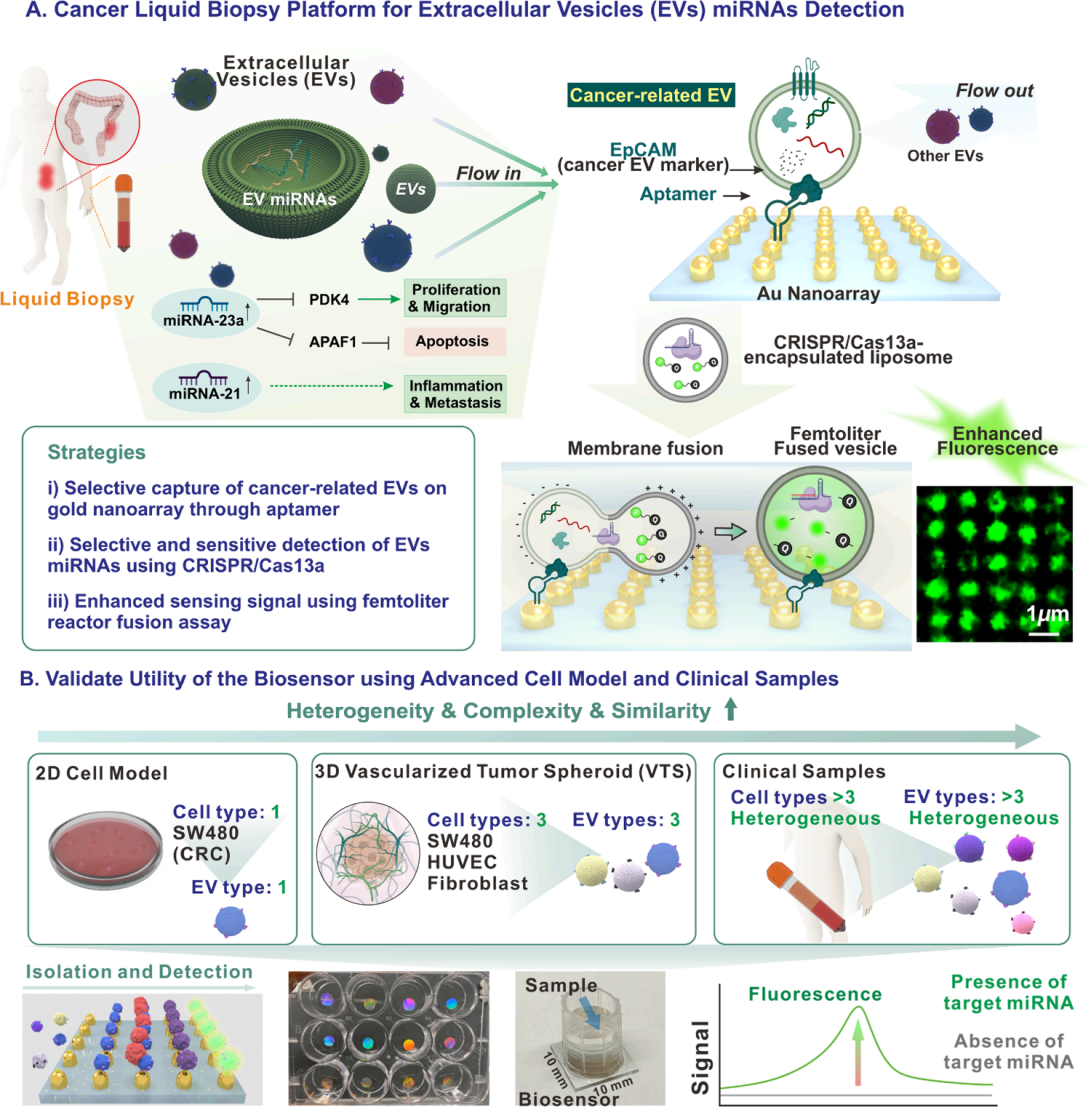
**Schematic diagram of the developed CRC liquid biopsy platform.** (A) Illustration of the liquid biopsy platform: cancer-associated EVs from CRC patient plasma are selectively captured by an EpCAM aptamer-functionalized gold nanoarray. These EVs subsequently fuse with CRISPR/Cas13a sensing probe-encapsulated liposomes, resulting in enhanced fluorescence recovery on the gold nanoarray. (B) Schematic representation of EV sources used to validate the platform, including EVs derived from SW480 2D cell cultures, 3D VTS model and clinical samples from patient blood plasma. The bottom panel illustrates the liquid biopsy platform, demonstrating the integrated process of selective EV isolation and detection on a single substrate. It also shows a real optical images of the gold nanoarray substrates in a 12-well plate with a holographic effect and a single biosensor configuration on a 10 mm × 10 mm glass substrate with samples added to the chamber. Fluorescence recovery indicates the presence of target EV miRNAs, while the absence of target miRNAs results in no detectable fluorescence signal. Schematic created with BioRender.com.

Despite the advantages of using EV miRNAs as biomarkers in liquid biopsy, several challenges hinder their effective application in cancer diagnosis. Traditional EV isolation methods, such as ultracentrifugation and size exclusion chromatography, lack the specificity to isolate cancer-related EVs from the complex milieu of biological fluids.^34–36^ The heterogeneity of EV populations, encompassing variations in size, molecular content, and cellular origin, further complicates the selective isolation and detection of disease-specific EVs. Additionally, while EVs carry a wide range of miRNAs, conventional liquid biopsy methodologies often fail to deliver the required selectivity and sensitivity, particularly when working with limited sample volumes. Established miRNA detection techniques, such as quantitative reverse transcription polymerase chain reaction (RT-qPCR) and next-generation sequencing (NGS), involve intricate and labor-intensive processes, including EV lysis, RNA extraction, and amplification.^37,38^ These approaches necessitate specialized instrumentation and substantial technical expertise, posing significant barriers to their routine clinical implementation. Furthermore, the EV lysis process, which disrupts the lipid bilayer to release miRNA cargo, may reduce miRNA yield and cause degradation due to the loss of the protective membrane. Therefore, maintaining EV integrity throughout the detection process is critical to preserving their biomolecular contents.

In parallel, in recent years, clustered regularly interspaced short palindromic repeats (CRISPR)-based diagnostics have gained traction for detecting viral DNA and RNA in clinical samples,^39^ as exemplified by platforms like DETECTR,^40^ HOLMES,^41^ SHERLOCK,^42^ SHERLOCK v2,^42^ and LEOPARD.^43^ Among these, CRISPR/Cas13a has been identified as a highly specific and sensitive tool for RNA detection.^44,45^ Selective detection using this system can be achieved by designing CRISPR RNAs (crRNAs) complementary to target miRNAs. Upon binding to the target miRNAs, the Cas13a/crRNA ribonucleoprotein (RNP) complex becomes activated, initiating collateral cleavage of quenched fluorescent RNA reporters.^46^ This cleavage generates a measurable fluorescence signal, enabling sensitive detection. The collateral cleavage activity of CRISPR/Cas13a is a self-amplified process, which significantly enhances the sensitivity of miRNA detection.^47,48^ However, many CRISPR-based detection systems necessitate nucleic acid preamplification to achieve sufficient signal levels, introducing additional complexity to the workflow and increasing the risk of false-positive results.^49,50^ By optimizing these systems, this study aims to overcome existing limitations and advance the application of CRISPR/Cas technology in liquid biopsy for cancer diagnostics.

To address critical limitations in cancer liquid biopsy and propel the advancement of CRISPR-based diagnostics, we have developed an integrated liquid biopsy platform that synergistically combines three nanobiotechnologies to enhance the detection of EV-associated miRNAs. Our advanced biosensing platform is designed to improve the specificity, sensitivity, and efficiency of cancer-related EV miRNAs detection while validating its clinical relevance for cancer diagnosis [Figure 1]. *First*, we utilized nanofabrication techniques to create gold nanoscale structures on a substrate using laser interference lithography (LIL).^51–53^ These structures were functionalized with EpCAM aptamers to selectively capture cancer-specific EVs from complex biological EV populations.^54–56^ The nanopatterned array effectively addresses EV heterogeneity by leveraging aptamer-antigen interactions, enabling the selective isolation of cancer-specific EVs from mixed biofluid samples. This strategy not only increases the specificity of EV capture but also provides a larger surface area for efficient EV immobilization. The integrated design of the gold nanoarray enables simultaneous enrichment and detection of cancer-specific EVs on a single chip, streamlining the overall workflow and reducing procedural complexity. Furthermore, the highly ordered and precisely controlled nanoarray architecture ensures reproducibility and batch-to-batch consistency, both of which are essential attributes for reliable clinical diagnostics and effective translation into clinical settings. *Second*, the platform integrates CRISPR/Cas13a-based RNA detection technology to enable highly selective and sensitive detection of specific EV miRNAs, such as miRNA-21 and miRNA-23a.^57–59^ *Lastly*, we applied liposomes to deliver CRISPR/Cas13a sensing probes into EVs via lipid membrane fusion, forming nanoscale reactors for improved fluorescence signal detection.^60–63^ These synthetic liposomes serve as carriers for the CRISPR/Cas13a detection probes, ensuring the structural integrity of EVs and protecting miRNAs from degradation through the fusion mechanism.

To validate our nanoliquid biopsy platform, we conducted a proof-of-concept study to demonstrate its sensitivity and selectivity for colorectal cancer (CRC) diagnosis. In the context of CRC EVs, miRNA-21 and miRNA-23a are well-established as upregulated biomarkers.^58,59,64^ The rationale for including both miRNA-21 and miRNA-23a lies in their complementary diagnostic value. miRNA-21 is one of the most widely studied miRNAs and is frequently upregulated in various cancers and inflammatory conditions,^65^ thereby enhancing detection robustness. It targets several tumor-suppressor genes, including PTEN (phosphatase and tensin homologue), PDCD4 (programmed cell death protein 4), RECK (reversion-inducing cysteine-rich protein with Kazal motifs), STAT3 (signal transducer and activator of transcription 3), and KRIT1 (Krev interaction trapped protein 1), among others.^66–68^ In addition, elevated levels of miRNA-21 are strongly associated with CRC metastasis and a pro-inflammatory tumor microenvironment.^65^ MiRNA-23a has been reported to exhibit higher specificity for colorectal cancer. MiRNA-23a has been shown to suppress pyruvate dehydrogenase lipoamide kinase isozyme 4 (PDK4), a negative regulator of CRC proliferation, thereby promoting tumor growth.^69^ It also influences cancer cell apoptosis through the APAF-1/caspase-9 apoptotic pathway.^70^ Together, the inclusion of miRNA-21 and miRNA-23a enables broader and more accurate detection of colorectal cancer by combining a general inflammation-associated marker with a cancer-specific one, strengthening the diagnostic and monitoring potential of the platform. We evaluated the platform using EVs derived from three distinct models: (i) a two-dimensional (2D) SW480 cell model, (ii) a three-dimensional (3D) vascularized tumor spheroid (VTS) model, and (iii) clinical patient plasma samples [Figure 1B]. The 2D SW480 cell model provided a basic in vitro representation of CRC cells, offering a controlled system for the initial assessment of EV capture and miRNA detection. However, this model exclusively generates EVs from SW480 cells, limiting its relevance for selectively evaluating the platform’s ability to capture cancer-specific EVs from complex biological samples. Furthermore, the absence of interactions with other cell types and extracellular matrix (ECM) components makes it challenging to replicate the tumor microenvironment or accurately reflect in vivo miRNA profiles.^71–73^ To overcome these limitations, we further developed a 3D VTS model incorporating multiple cell types and ECM components to better mimic the in vivo tumor microenvironment. This advanced model allowed for a more accurate evaluation of the platform’s ability to capture cancer-specific EVs and detect relevant miRNA contents, providing a robust system for testing biosensor functionality. Finally, clinical validation was performed using plasma samples from CRC patients and noncancerous controls. This step confirmed the platform’s clinical applicability and diagnostic potential. The sequential validation strategy, progressing from a simple 2D cell model to a more complex 3D VTS model and ultimately to clinical plasma samples, demonstrates a comprehensive approach. This progression increases the heterogeneity and complexity of EV samples, closely approximating those found in clinical settings. As a result, our liquid biopsy platform has been effectively validated for its clinical relevance and reliability in CRC diagnosis.

*In summary*, the advanced biosensing platform represents system-level engineering optimized across the entire liquid biopsy workflow on a highly ordered and scalable gold nanoarray substrate, from selective EV enrichment to sensitive detection, tailored specifically for clinical translation. Importantly, this platform has been rigorously validated across multiple biologically relevant models, achieving comprehensive clinical applicability and robustness previously unachieved in the field. By synergizing three key innovations, EpCAM aptamer-functionalized gold nanoarrays for cancer-specific EV capture, CRISPR/Cas13a-based RNA sensing for sensitive and selective miRNA detection, and liposome and EV fusion-mediated nanoscale reactors for signal amplification, this platform addresses critical shortcomings in conventional methodologies, such as low specificity in EV isolation, RNA degradation during extraction, and insufficient sensitivity in biomarker detection. These advancements collectively enhance the accuracy of cancer diagnosis and longitudinal monitoring, offering a robust alternative to existing techniques. While validated using established biomarkers (EpCAM, miRNA-21 and miRNA-23a) for colorectal cancer detection, the platform’s modular architecture that are characterized by substitutable aptamers for biomarker-specific EV capture, customizable CRISPR probes for target RNA detection, and adjustable gold nanoarray, positions it as a broadly applicable and versatile diagnostic tool. This flexibility allows our platform to be adapted for detecting distinct disease-specific biomarkers across a wide spectrum of clinical conditions, including various cancer types and neurodegenerative disorders such as Alzheimer’s disease. We anticipate that this technology will become a transformative asset in clinical practice, bridging the gap between laboratory research and real-world applications. By facilitating earlier and more precise disease detection, it may help improve patient outcomes and guide personalized therapeutic strategies.

## Results and Discussion

### Design and Fabrication of CRISPR/Cas13a-Enabled Ultrasensitive and Specific Detection of Colorectal Cancer-Associated miRNAs

MiRNAs are short, noncoding RNA molecules that function as pivotal gene expression regulators, acting as oncogenes or tumor suppressors to modulate critical cellular processes.^74^ Dysregulated miRNA expression, whether upregulated or downregulated, is strongly implicated in driving hallmark features of cancer, including uncontrolled proliferation, metastatic spread, and apoptosis.^75^ These characteristics make miRNAs valuable biomarkers for cancer detection and monitoring. In this study, we leveraged the CRISPR/Cas13a system to target two oncogenic miRNAs, miRNA-21 and miRNA-23a, which are consistently overexpressed in CRC and correlate with disease progression.^64^ The CRISPR/Cas13a platform operates through a sequence-specific mechanism: a crRNA guide directs the Cas13a endonuclease to bind complementary target miRNAs. Upon recognition, the Cas13a/crRNA complex undergoes a conformational activation, unleashing its collateral cleavage activity. This activity indiscriminately degrades nearby RNA reporters engineered with a fluorophore-quencher pair. Cleavage of the reporter physically separates the fluorophore from its quencher, thereby restoring fluorescence emission. The resulting fluorescence signal, proportional to the target miRNA concentration, provides a quantifiable readout, enabling ultrasensitive detection. This approach not only enhances specificity through crRNA-guided targeting, but also amplifies signal, thus improving sensitivity, via Cas13a’s collateral activity.

To evaluate the precision and effectiveness of the CRISPR/Cas13a biosensing system, trans-cleavage activity was evaluated under various conditions, as illustrated in [Figure 2A], including: (i) activated CRISPR/Cas13a with RNA reporter, (ii) nonactivated CRISPR/Cas13a with RNA reporter (where target miRNA was absent), (iii) nonactivated CRISPR/Cas13a without crRNA, (iv) RNA reporter alone, and (v) activated CRISPR/Cas13a with a DNA reporter. Fluorescence intensity at 517 nm was measured for each condition to assess trans-cleavage activity. As shown in [Figure 2B], fluorescence recovery occurred only when all required components, Cas13a enzyme, crRNA, target miRNA-23a, and RNA reporter, were present (condition (i). No fluorescence was observed in other conditions, confirming that trans-cleavage was specific to RNA reporters and did not interfere with DNA. Further validation of collateral activity was performed through gel electrophoresis [Figure S1]. Activated CRISPR/Cas13a complexes cleaved RNA reporters, resulting in a downward shift in the RNA bands compared with samples containing only RNA reporters. The results showed that the activated CRISPR/Cas13a system fragmented the RNA reporters. The detection sensitivity of the CRISPR/Cas13a system was evaluated by measuring fluorescence intensity over time with varying concentrations of miRNA-23a, ranging from 100 nM to 100 fM [Figure 2C]. Fluorescence intensity increased with higher miRNA concentrations until a stable signal was achieved. A highly linear relationship was observed between miRNA concentration and fluorescence intensity within the range of 1 pM to 1 nM (R^2^ = 0.9981) at 25 min [Figure 2D]. The limit of detection (LOD) for miRNA-23a was determined to be 1 pM, calculated using LOD = 3s/b (where s is the standard deviation of the background signal and b is the linear regression slope). The specificity of the CRISPR/Cas13a detection system was also validated. The crRNA was specifically designed to be complementary to miRNA-23a, ensuring that only miRNA-23a could activate the CRISPR/Cas13a complex, while miRNA-21 would not trigger activation [Figure 2E]. A second crRNA targeting miRNA-21 was also designed using a spacer sequence specific to miRNA-21. Experiments were conducted using both crRNAs and their respective target miRNAs. As shown in [Figure 2F], fluorescence recovery occurred only when the CRISPR/Cas13a complex was activated by its specific target miRNA and crRNA. These results confirm the specificity and sensitivity of the CRISPR/Cas13a-based detection system for miRNA-21 and miRNA-23a, demonstrating its suitability for reliable EV miRNA detection in the following liquid biopsy applications.

**Figure 2:**
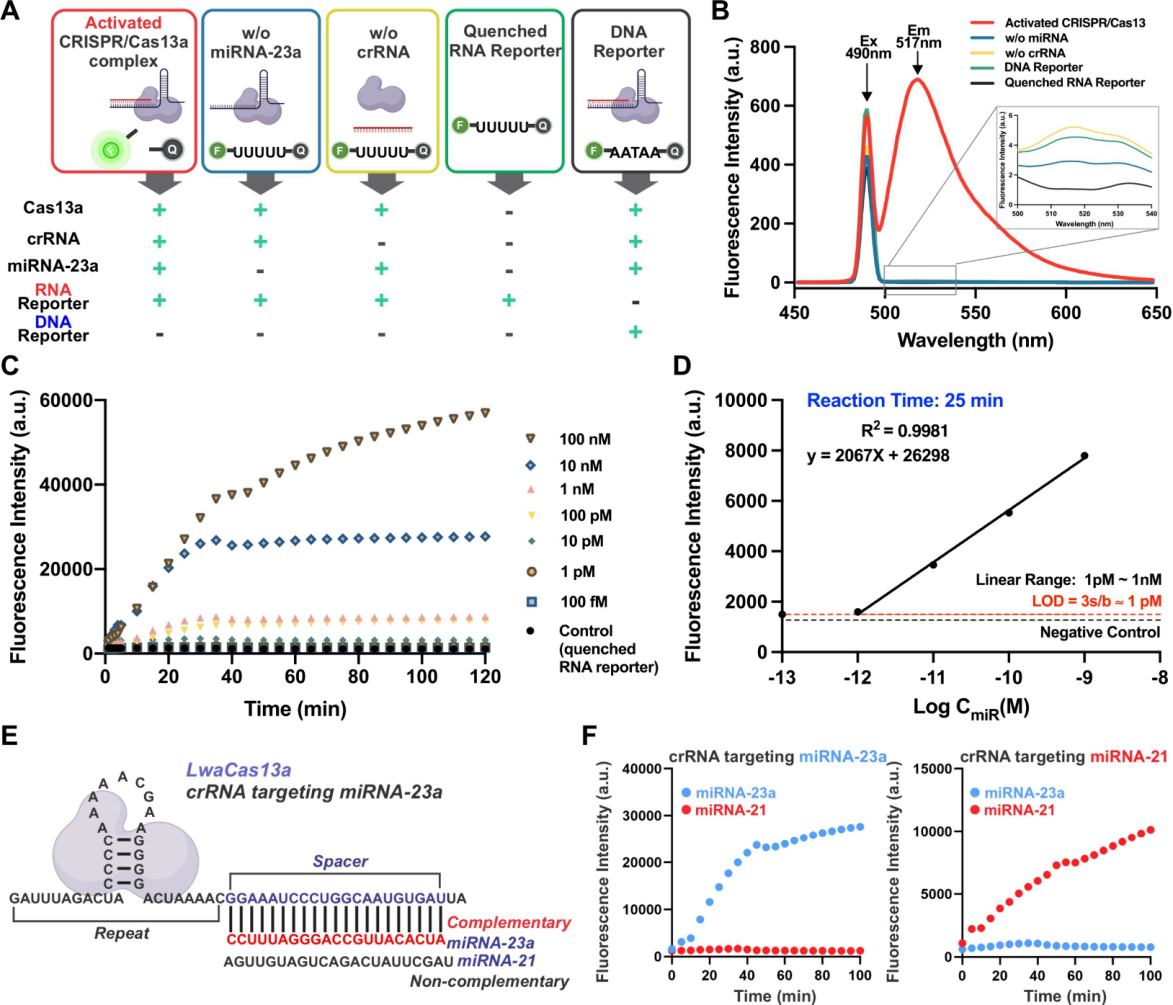
**Detection of miRNAs using a CRISPR/Cas13a sensing system.** (A) Schematic representation of the CRISPR/Cas13a-based RNA detection mechanism, highlighting its collateral cleavage activity. (B) Fluorescent response of the CRISPR/Cas13a system under varying conditions as illustrated in (A). (C) Fluorescent intensity profile versus reaction time demonstrating the detection of miRNA-23a at varying concentrations range from 100 nM to 100 fM. (D) Linear detection curve of miRNA-23a at a reaction time of 25 min using the CRISPR/Cas13a system. (E) Schematic illustrating the selective detection process, where the complementary target sequence is recognized by crRNA, activating CRISPR/Cas13a collateral cleavage. (F) Selective detection of miRNA-23a using crRNA designed for miRNA-23a (Left). Specific detection of miRNA-21 using crRNA complementary to miRNA-21 (Right). Schematic created with BioRender.com.

### Fusion-Driven Nanoscale Reactors: CRISPR/Cas13a-Mediated Amplification of miRNA Signals in Colorectal Cancer-Derived EVs

EV miRNAs are pivotal in liquid biopsy for noninvasive disease monitoring, but conventional lysis-dependent extraction methods suffer from low yields and RNA degradation, limiting sensitivity.^76,77^ To overcome this, we developed a lysis-free fusion strategy [Figure 1A], where EVs fuse with CRISPR/Cas13a sensing probe-loaded liposomes, forming *femtoliter nanoreactors*. This method preserves EV integrity and amplifies detection signal by concentrating target-probe interactions in nanoscale reactors, thereby amplifying fluorescence signal through confining CRISPR/Cas13a-mediate trans-cleavage in highly localized vesicles. EVs used to validate the assay were isolated from SW480 cells-conditioned media via an optimized aqueous two-phase system (ATPS)^78–80^ [Figure S2], which outperforms traditional polyethylene glycol (PEG)-based precipitation methods in purity. Unlike PEG-based precipitation, which cannot effectively separate EVs from lipoproteins, the ATPS method efficiently partitions lipoproteins into the PEG phase while enriching EVs in the dextran phase, reducing contaminants and minimizing interference in downstream analysis. This selective separation is driven by differences in the hydrophilicity of PEG and dextran to different biomolecules, enabling the isolation of high-purity EVs for improved assay validation. Dynamic light scattering (DLS) analysis confirmed that PEG-only isolation yielded vesicles with two distinct peaks at ∼ 30 nm (lipoproteins) and ∼ 100–200 nm (EVs) [Figure S3A]. In contrast, ATPS isolation shows particles in the PEG phase primarily at ∼ 30–40 nm (lipoproteins) and particles in the dextran phase at ∼ 100–200 nm (EVs) [Figure S3B **& C**]. Transmission electron microscopy (TEM) further validates the separation efficiency, showing coexisting lipoproteins and EVs in the PEG-only method [Figure S3A] but distinct populations in ATPS-separated phases, with lipoproteins mainly in the PEG phase and EVs predominantly in the dextran phase [Figure S3B **& C**]. Western blot analysis confirmed the identity of the isolated EVs, demonstrating the presence of β-actin (housekeeping marker) in both EVs and parent cells SW480, while CD63 and CD81, common EV markers, were detected exclusively in EVs [Figure 3A]. Liposomes were synthesized using a mixture of lipids [Table S1] via thin-film hydration and extrusion, as described in the Methods section. The zeta potential analysis revealed that EVs carried a negative charge at −8.46 mV, while liposomes were positively charged at 19.4 mV [Figure S5]. The fusion of these two vesicles was facilitated by the electrostatic force between their opposing charges. The fusion between EVs and liposomes was characterized using TEM, nanoparticle tracking analysis (NTA), and Förster resonance energy transfer (FRET) assays [Figure 3 **B-E**]. TEM images [Figure 3B] revealed the typical morphology of EVs and liposomes. After fusion, vesicle size increased. NTA also measured the particle size distribution and number for each type of vesicle, as detailed in Figures 3C. EVs and liposomes were mixed at a 1:1 number ratio and incubated at 37 °C for 1 h to facilitate fusion. NTA showed that the average size of EVs was approximately 107.2 nm, and the liposomes had a mean size of 127.2 nm. Following fusion, the size of the resulting vesicles increased to 233.3 nm, confirming the successful fusion of EVs and liposomes. In addition, DLS was performed to validate the size distribution and surface charge of the particles, further confirming the fusion process [Figure S4]. FRET assays were then used to assess the fusion efficiency between EVs and the synthetic fusogenic liposomes. Liposomes were doped with a FRET pair, BODIPY cholesterol (donor), and Lissamine Rhod B DHPE (acceptor) [Figure 3D]. Before fusion, these two fluorophores reside in close proximity within the lipid bilayer, allowing energy transfer from the donor to the acceptor. Upon excitation at 488 nm, strong acceptor emission at 590 nm was observed, indicating efficient FRET activity. Following fusion with EVs, the lipid bilayers merge, leading to a redistribution and increased spatial separation between donor and acceptor molecules. As a result, the FRET efficiency decreases, and donor emission at 508 nm is predominantly observed [Figure 3E], indicating membrane fusion and probe dilution. To validate that the observed FRET change is specifically due to membrane fusion rather than lipid exchange or aggregation, a nonfusogenic liposome (lipid composition detailed in Table S1) was synthesized and used as a negative control. These liposomes lack fusogenic components and were incubated with EVs under identical conditions (37 °C for 1 h). The resulting FRET emission profiles [Figure S6] showed a reduced shift compared to the fusogenic liposomes. To quantify fusion efficiency, the FRET ratio was calculated using the formula:

$$FRETratio={FI}_{acceptor}/{FI}_{donor}$$

where *FI*_*acceptor*_ and *FI*_*donor*_ represent the fluorescence intensities at 590 and 508 nm, respectively. The normalized FRET ratio change (ΔFRET) was then defined as

$$\Delta FRET=\left(FRET_{before}-FRET_{after}\right)/FRET_{before}$$

where *FRET*_*before*_ and *FRET*_*after*_ refer to the FRET ratios before and after fusion. The calculated Δ*FRET* for fusogenic liposomes and EVs was 0.97, indicating nearly complete disruption of FRET and highly efficient fusion between EVs and liposomes. In contrast, the Δ*FRET* for nonfusogenic liposomes and EVs was 0.23, reflecting limited fusion activity. These results collectively demonstrate that the fusogenic liposomes can effectively fuse with EV membranes, enabling the delivery of encapsulated CRISPR/Cas13a sensing probes directly into the EV lumen. This fusion mechanism is critical to the function of the developed detection platform, as it preserves EV integrity while enhancing localized sensing sensitivity by forming femtoliter-scale reaction compartments.

**Figure 3:**
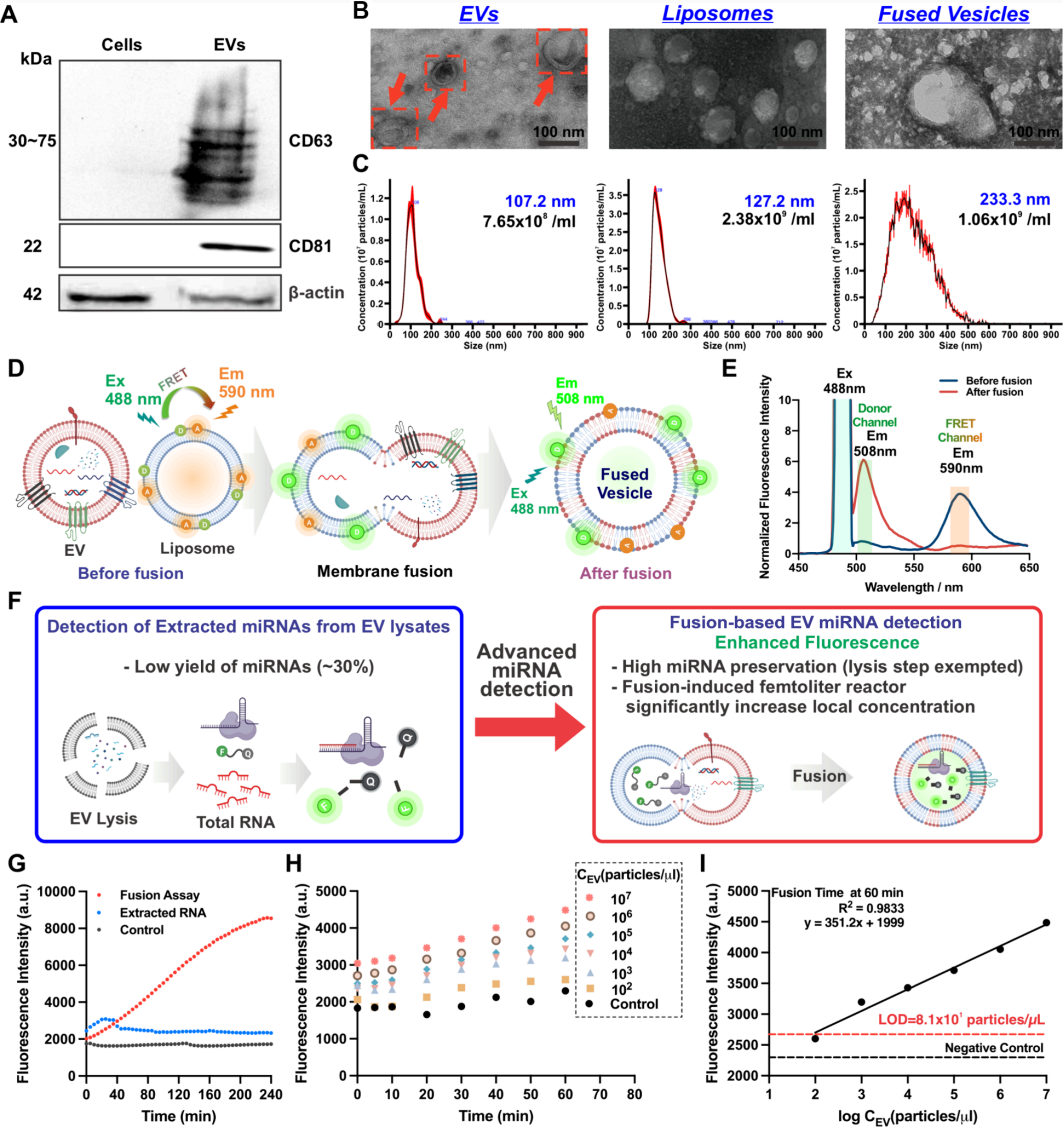
**Characterization of EVs, synthetic liposomes, and fused vesicles, and miRNA detection via a fusion assay.** (A) Western blot analysis showing EV-specific markers (CD63 and CD81) present in EVs derived from SW480 cells, but absent in SW480 cells. (B) Representative TEM images of EVs, liposomes, and fused vesicles, respectively. (C) Size distribution and particle concentration of EVs, liposomes, and fused vesicles measured using NTA. (D) Schematic illustration of the FRET assay confirming fusion activity between EVs and liposomes. (E) FRET assay results demonstrate an increased FRET donor signal (508 nm) and decreased FRET acceptor signal (590 nm), indicating successful membrane fusion and the separation of FRET dyes following the interaction of FRET-labeled EVs and liposomes. (F) Schematic representations of direct detection of extracted miRNAs from EVs and the fusion-based assay, respectively. (G) Fluorescent response of EV miRNA-23a detection using the fusion assay compared to extracted RNA versus reaction time. (H) Fluorescent response of EV miRNA-23a detection at varying EV concentrations from 10^7^ particles/μL to 10^2^ particles/μL using the fusion assay. (I) Linear detection range and limit of detection of EV miRNA-23a using the fusion assay at a fusion time of 60 min in bulk. Schematic created with BioRender.com.

To assess the improved sensitivity of EV miRNA detection using the CRISPR/Cas13a fusion assay, fluorescence intensity was compared to that of direct detection of extracted total RNAs from EV lysates in bulk [Figure 3F]. The fluorescence measurements from both assays are shown in [Figure 3G]. The results demonstrated that, during the direct detection of RNA extracted from EV lysates using the CRISPR/Cas13a system, the fluorescence intensity increased steadily within the first 30 min and plateaued as the reaction reached completion. In contrast, the fusion-based method showed lower fluorescence intensity during the initial 40 min of the reaction, attributed to the time required for lipid mixing and membrane fusion to deliver the CRISPR/Cas13a sensing probes into EVs. However, beyond 40 min, the fluorescence intensity from the fusion-based method surpassed that of direct detection and continued to increase steadily for up to 4 h. At the 4-h mark, the fluorescence intensity increase relative to the extracted control background in the fusion method was 11.2 times higher than that of direct miRNA detection from extracted RNA, 6.2 folds higher at the 2-h mark, and 2.2 times higher at 1-h mark. This enhanced detection efficiency can be explained by two key factors: (i) the preservation of miRNA integrity within intact EVs, avoiding degradation caused by lysis and purification steps, and (ii) the spatial confinement of target miRNAs and CRISPR/Cas13a sensing probes within femtoliter-scale nanoreactors, which increases local concentrations and accelerates molecular interactions. In contrast, conventional methods relying on EV lysis risk miRNA degradation during vesicle disruption and disperse targets in bulk solution, leading to diluted analyte-probe interactions and reduced sensitivity.

To further assess the sensitivity of the fusion-based assay, fluorescence intensity was measured at varying EV concentrations [Figure 3H]. Higher EV concentrations resulted in higher fluorescence signals, demonstrating the assay’s sensitivity to changes in EV numbers. A standard curve was generated [Figure 3I], showing a linear relationship between fluorescence intensity and EV concentrations ranging from 10^2^ particles/μL to 10^7^ particles/μL. The fusion assay achieved a LOD of 8.1 × 10^1^ particles/μL, underscoring its high sensitivity. This study confirms CRISPR/Cas13a’s fusion-based method as a highly efficient way to detect miRNAs in EVs. This method significantly enhances miRNA detection sensitivity, reduces the required sample volume, and preserves miRNAs within intact EVs, making it a promising tool for liquid biopsy applications. Overall, by integrating the ATPS-based EV isolation method and fusion-driven nanoreactors, our platform improves the sensitivity, adaptability and reliability of EV miRNA analysis. However, the fusion assay in solution is limited to single-cell-derived EV miRNA detection, which may not be ideal for clinical diagnostics due to the heterogeneity of EVs present in biofluids.^81^ This limitation arises from the indiscriminate feature of the fusion process between liposomes and EVs, which does not differentiate between cancer-specific EVs and other EV subpopulations. Enhancing the selective detection of cancer-derived EVs within complex biological samples holds significant promise for advancing liquid biopsy technologies. To address this challenge, we introduce two synergistic innovations: (i) a VTS model that recapitulates the in vivo tumor microenvironment by coculturing cancer cells, fibroblasts, and endothelial cells, enabling the production of heterogeneous EVs reflective of clinical samples; and (ii) an aptamer-functionalized gold nanoarray engineered to selectively capture cancer-specific EVs via EpCAM-targeting aptamers, leveraging high-affinity interactions to isolate tumor-derived vesicles from mixed EV populations. Together, these strategies improve diagnostic specificity by resolving the heterogeneity of EV subpopulations while maintaining high sensitivity, thereby overcoming critical limitations in conventional EV-based cancer detection workflows.

### Advanced 3D Vascularized Tumor Spheroid Model: Generation, Functional Validation, and Clinical Relevance in CRC Diagnostics

EVs are indispensable mediators of intercellular communication and are emerging as clinically relevant biomarkers for liquid biopsy, as they encapsulate molecular signatures mirroring the pathophysiological state of their parent cells.^26,82^ While traditional 2D monocellular cultures offer simplicity and accessibility, EVs derived from these systems exhibit molecular and functional disparities compared to EVs isolated from patient biofluids. This discrepancy arises from the absence of critical cell-to-cell interactions and ECM signaling, that are key microenvironmental features that govern EV cargo sorting in vivo.^72,73,83,84^ To bridge this gap, advanced 3D culture systems have been developed to emulate the multicellular complexity of tumors, producing EVs with molecular profiles that closely resemble those of circulating EVs in cancer patients.^71–73^ To validate the clinical translatability of our liquid biopsy platform, we engineered a 3D VTS model, integrating cancer cells, stromal fibroblasts, and endothelial cells within a biomimetic ECM. This model recapitulates the dynamic cellular crosstalk and heterogeneity of in vivo tumors, enabling the generation of EVs that reflect clinical samples for robust diagnostic testing.

The VTS model comprises three cell types: SW480 colorectal cancer cells, lung fibroblasts (LFs), and human umbilical vein endothelial cells (HUVECs), embedded in a matrix composed of Matrigel and fibrin gel to mimic the ECM. In this biomimetic environment, cells are exposed to more extensive interactions with neighboring cells and the matrix, enabling growth in multiple dimensions. These interactions enhance cellular signaling and allow cells to exhibit more in vivo-like behaviors and responses.^71–73^ Consequently, EVs derived from the VTS system exhibit molecular characteristics more closely resembling EVs found in human biofluids. In addition to better mimicking the tumor microenvironment, EVs derived from the VTS model are a mixture of vesicles from three different cell types. This heterogeneity makes the model particularly valuable for studying the specific isolation of cancer-derived EVs from total EV populations.

The timeline for generating the VTS model is illustrated in [Figure 4A], with a detailed protocol provided in the Methods section. SW480 tumor spheroids (TSs) were created using a previously developed microwell-based protocol, which enables the generation of highly uniform spheroids in a high-throughput and efficient manner.^85^ Optical and fluorescent images of SW480 TSs in hydrogel microwells show an average size of 250 μm [Figure 4 **B** **& 4C**]. Scanning electron microscopy (SEM) images [Figure 4D] further confirm the size of individual spheroids, correlating with the optical imaging results. The generated SW480 TSs were tricultured with HUVECs and LFs, which served as vascular and supporting cells, respectively, and embedded in fibrin gel as ECM in a specially designed chip. Over 7 days of triculture, brightfield images revealed steady spheroid growth from 258 to 374 μm, along with the formation of a vascular network [Figure S7A **& B**]. On day 7, immunostaining showed evidence of vasculogenesis around the SW480 TSs [Figure 4E]. A cross-sectional 3D projection image [Figure S8] indicated that vascular structures also developed within the lumen of the SW480 TSs, confirming the successful establishment of a 3D vascularized cell culture model.

**Figure 4:**
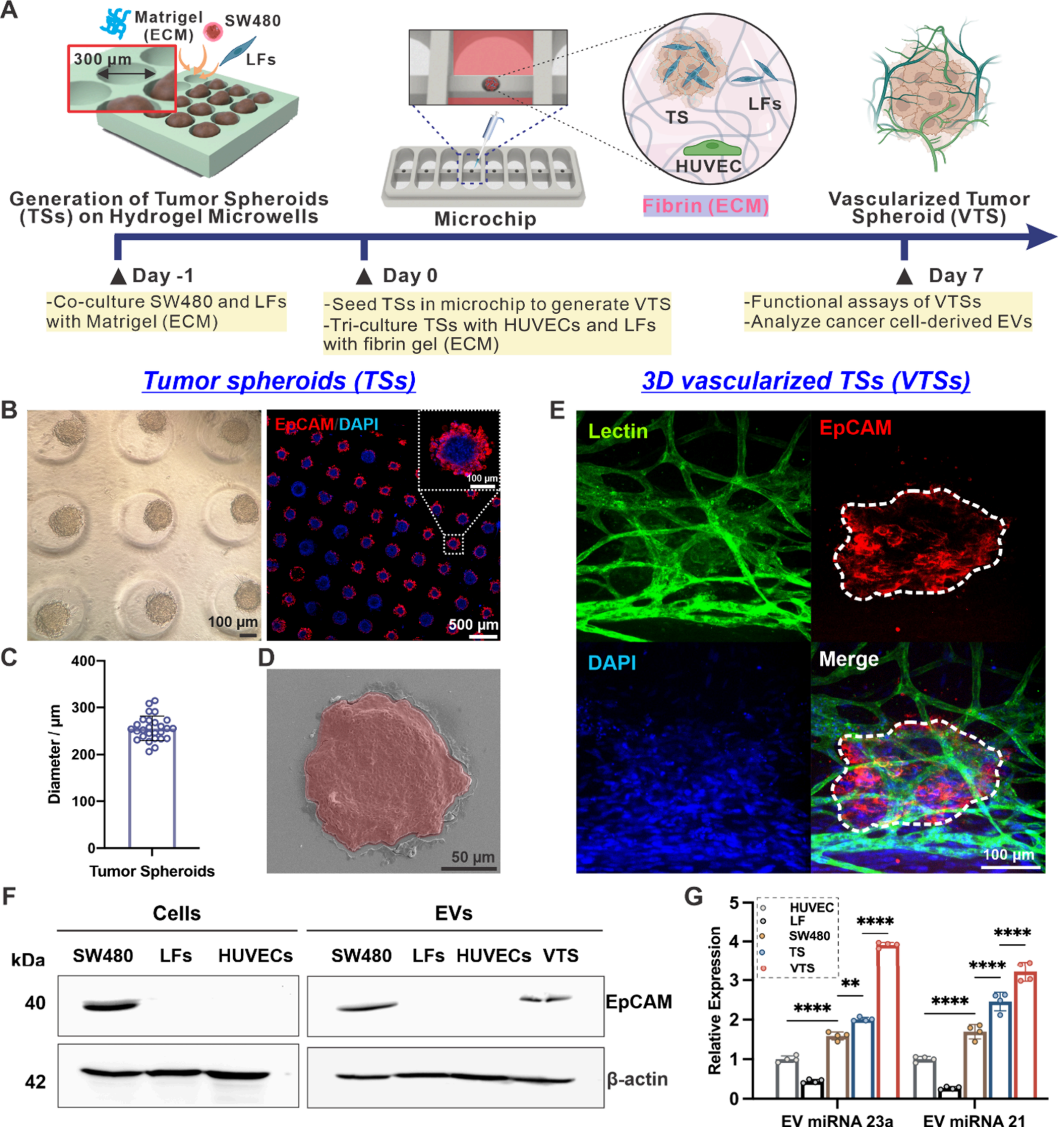
**Characterization of VTS and EVs derived from VTS model.** (A) Schematic workflow illustrating the fabrication of tumor spheroids and vascularized tumor spheroids on chip. (B) Optical images of tumor spheroids formed within the hydrogel microwell array (Left). Confocal images of tumor spheroids formed in hydrogel microwells, stained for EpCAM (red) and nuclei (DAPI, blue) (Right). (C) Size distribution of hydrogel microwells, with an average size of 250 μm (*n* = 27, data represent mean ± SD). (D) Representative SEM image of an individual tumor spheroid with red pseudocoloring. (E) Representative confocal images of VTS, stained for Lectin (green), EpCAM (red) and nuclei (DAPI, blue). (F) Western blot analysis of cells, including SW480, LFs, and HUVECs and EVs derived from 2D cell cultures (SW480, LFs, and HUVECs) and 3D VTS cell culture models. (G) RT-qPCR results showing the expression levels of miRNA-23a and miRNA-21 from EVs of different cells and culture conditions (*n* = 4, bar graph represents mean ± SD, by one-way ANOVA analysis, ** *p* < 0.01, **** *p* < 0.0001). Schematic created with BioRender.com.

Next, the molecular contents of EVs derived from the VTS model and other cell culture systems were analyzed. EpCAM, a biomarker commonly overexpressed in many carcinomas, was examined. Western blot analysis [Figure 4F] confirmed that EpCAM was present in SW480 cells and SW480 cells-derived EVs, and the absence of EpCAM in LFs and HUVECs, both cell and EVs. These results confirm that the EpCAM signal detected in VTS-derived EVs originates from SW480 cells, indicating that EpCAM can serve as a potential biomarker to differentiate cancer-specific EVs from other EVs. To investigate EV RNA profiles, RT-qPCR was performed to analyze miRNA-23a and miRNA-21 expression across different cell culture conditions [Figure 4G]. EVs were collected and processed from 2D-cultured SW480 cells, HUVECs, LFs, 3D SW480 TSs, and 3D VTS systems. Distinct expression profiles of miRNA-23a and miRNA-21 were identified across the tested models. EVs isolated from the 3D VTS system showed markedly higher levels of these miRNAs compared to EVs derived from traditional 2D monocultures or simpler 3D-systems without vascular structures. These results underscore that the VTS model which recapitulates complex cell–cell and cell-matrix interactions within a biomimetic microenvironment can potentially generates RNA cargo profiles that more closely mirror those of EVs isolated from patient-derived clinical samples. The RT-qPCR results indicate that the VTS system offers a more physiologically relevant in vitro model for studying EV biomarkers in liquid biopsy diagnostics.

### Specific Capture and Ultrasensitive Detection of Colorectal Cancer EV miRNAs via Aptamer-Functionalized Gold Nanoarray and CRISPR/Cas13a Fusion Assay

The VTS model was engineered to recapitulate the heterogeneity and biomimicry of clinical tumor microenvironments, incorporating SW480 cells, HUVECs, and LFs to generate EVs reflective of in vivo cellular crosstalk. We integrated an aptamer-functionalized gold nanoarray into the platform to isolate cancer-derived EVs from this heterogeneous EV population. The nanoarray, fabricated via LIL and electrochemical deposition, features a 10 mm × 10 mm surface with periodic gold nanoscale patterns that maximize aptamer density and binding efficiency. These nanostructures enhance EV capture through high-affinity EpCAM aptamer-antigen interactions while ensuring signal reproducibility via uniform spatial alignment. EpCAM aptamers selected for their specificity to tumor-derived EVs were covalently immobilized on the gold surface using thiol-gold self-assembled monolayers (SAMs), enabling selective enrichment of cancer EVs over nontumor vesicles. This dual strategy (VTS + nanoarray) bridges the gap between in vitro EV heterogeneity and clinically biomarker detection, ensuring precise profiling of oncogenic miRNAs like miRNA-21 and miRNA-23a. This configuration ensures that EV-liposome fusion occurs only with the captured cancer-specific EVs, enhancing the sensitivity and selectivity of miRNA detection [Figure 5A].

**Figure 5:**
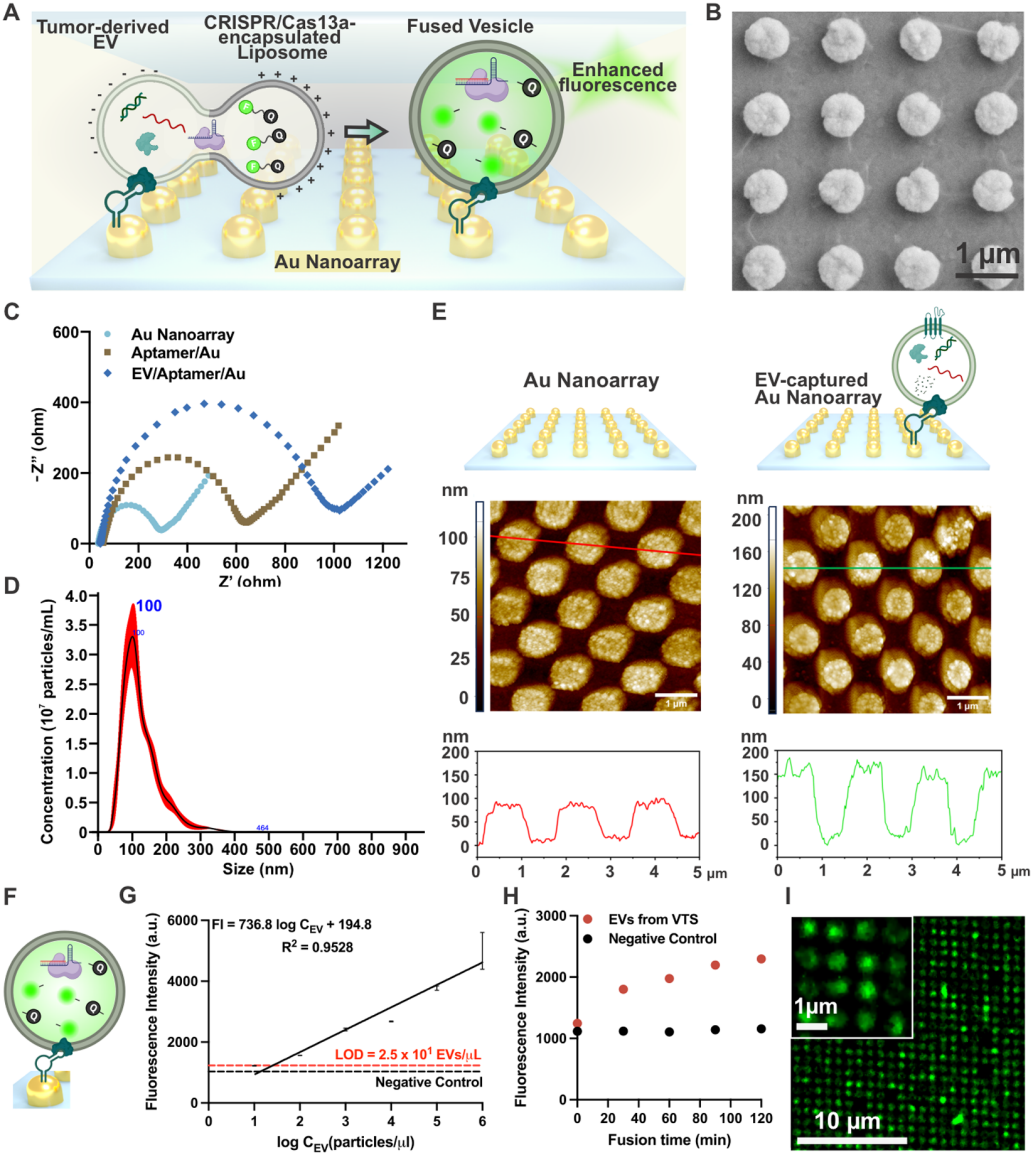
**Isolation and detection of cancer-related EVs on a gold nanoarray.** (A) Schematic diagram of the selective detection of cancer-related EVs on a gold nanoarray platform by fusion assay. (B) SEM images showing the gold nanoarray structure, scale bar 1 μm. (C) EIS Nyquist plots of the gold nanoarray, EpCAM aptamer-modified gold nanoarray, and EV-captured gold nanoarray. (D) NTA analysis of EVs derived from VTS. (E) AFM images and height profiles of the gold nanoarray before (left) and after (right) EV capture, scale bar 1 μm. (F) Schematic diagram illustrating fluorescence recovery during the fusion-based EV miRNA detection using CRISPR/Cas13a sensing probes. (G) Linear detection of SW480-derived EV miRNA-23a over the gold nanoarray by fusion assay (*n* = 4, data represent mean ± SD). (H) Fluorescence detection of VTS-derived EV miRNA-23a on the gold nanoarray by fusion assay. (I) Representative confocal fluorescence image of EV miRNA-23a detection by fusion assay on the gold nanoarray. Schematic created with BioRender.com.

LIL was used to fabricate nanosized holes in a thin layer of a photoresist (PR) [Figure S9], followed by gold electrochemical deposition within these holes, producing uniform 600 nm diameter gold nanoarrays, as confirmed by SEM imaging [Figure 5B]. To investigate the optimal gold nanostructure size for maximizing sensing performance, gold nanoarrays with feature sizes of 300, 600, and 1000 nm were fabricated using LIL by adjusting the incident angle. As a control, a flat gold substrate was prepared via gold electrochemical deposition on ITO glass without periodic nanostructures. SEM images of all four substrates [Figure S12A] confirmed that gold nanoarrays with distinct and uniform feature sizes were successfully fabricated using the LIL method. SW480-derived EVs, isolated from the same batch, were then used in fusion assays to assess the effect of gold nanostructure size on detection performance. The resulting fluorescence signals [Figure S12B] demonstrated that the 600 nm gold nanoarray produced the highest fluorescence intensity and exhibited statistically significant enhancement for both miRNA-21 and miRNA-23a detection compared to the 1000 nm, 300 nm nanoarrays, and the flat gold control. Substrates featuring periodic gold nanostructures outperformed the flat gold substrate, as the highly ordered nanostructures provide a larger surface area for EV capture, thereby enhancing detection. However, the 300 nm nanoarray exhibited a reduction in signal intensity despite its higher surface area. This was attributed to the excessive density of nanostructures, which likely introduced steric hindrance that hindered efficient fusion between liposomes and EVs. These findings demonstrate that an appropriately sized gold nanostructure is critical for balancing EV capture efficiency and fusion dynamics, ultimately enhancing fluorescence detection performance. EpCAM aptamers were then immobilized on the gold nanoarrays via thiol-gold interactions. Electrochemical impedance spectroscopy (EIS) [Figure 5C] showed a significant increase in impedance with aptamer modification and further increases after EV capture, confirming successful immobilization at each step. Cyclic voltammetry (CV) measurements [Figure S10] further validated the process: currents increased drastically after gold deposition due to the excellent conductivity of gold, decreased slightly after aptamer functionalization due to reduced electron accessibility, and decreased further after EV capture as electron accessibility to the surface was reduced, indicating successful surface modifications at each step. Atomic force microscopy (AFM) imaging [Figure 5E] confirmed EV capture on the aptamer-functionalized gold nanoarray. Prior to EV capture, the height of the bare gold nanoarray ranged from 68.2 to 100.7 nm, with an average of 85.0 nm. Following EV immobilization, the measured height increased to a range of 137.6 to 184.7 nm, with an average of 155.9 nm. The resulting height difference corresponds to captured vesicles with diameters ranging from 36.9 to 116.5 nm, as illustrated in Figure S13A, which are well within the physiological size range of extracellular vesicles [Figure 5D]. This observation confirms that the EVs remain structurally intact upon capture and that the process does not induce significant deformation or degradation, which is essential for downstream molecular profiling and accurate biosensing. In addition, to estimate the capture efficiency, NTA was performed before and after incubation of EV samples with gold nanoarray using. A 100 μL EV suspension at a concentration of 1.99 × 10^9^ particles/mL was incubated with the substrate. Following capture, the unbound EV concentration in the supernatant was measured as 1.13 × 10^9^ particles/mL [Figure S13B **].** This corresponds to an estimated 8.6 × 10^7^ EVs captured on a single nanoarray substrate. These results collectively confirm that our platform enables effective EV capture while maintaining vesicle integrity and physiologically relevant size distributions. After confirming successful EV capture, the fusion assay was performed with SW480-derived EVs captured on the nanoarray [Figure 5F]. Fluorescence intensity data [Figure 5G] showed a LOD of 2.5 × 10^1^ particles/μL with a linear detection range from 10 to 10^6^ EV particles/μL, demonstrating greater sensitivity than the LOD of 8.1 × 10^1^ particles/μL observed in the bulk fusion assay [Figure 3H]. The improvement in detection sensitivity observed on the gold nanoarray platform can be attributed to several key factors. First, the spatial confinement of captured EVs plays a crucial role in facilitating efficient and specific fusion. On the nanoarray, EVs are selectively immobilized using EpCAM-specific aptamers, ensuring that fusion occurs precisely at the surface-bound EVs. This controlled spatial arrangement enables highly efficient and targeted fusion with CRISPR/Cas13a-loaded liposomes. In contrast, in the bulk solution assay, fusion events occur randomly, leading to lower fusion efficiency and reduced detection sensitivity. Second, the enrichment of reaction components at the nanoarray surface enhances detection sensitivity. Conversely, in bulk solution, the fused vesicles are dispersed, leading to dilution of the fluorescence signal and reduced sensitivity. Fluorescence intensity over time [Figure 5H] revealed that fluorescence from VTS-derived EVs increased continuously while the negative control showed minimal change, confirming that the nanopattern-based fusion assay effectively facilitates CRISPR/Cas13a-based EV miRNA detection. Finally, [Figure 5I] and [Figure S11] show confocal microscopy images of the fluorescent signal from the detected EVs. Before fusion, no fluorescence was observed, but after fusion and cleavage of the quenched RNA reporters, green fluorescence of RNA reporters appeared on the nanoarrays, confirming successful EV-liposome fusion and activation of the CRISPR/Cas13a sensing system. Collectively, experiments employing EVs derived from the 3D VTS model and the aptamer-functionalized gold nanoarray validated the platform’s ability to specifically identify and isolate heterogeneous EV subpopulations, including cancer-derived vesicles, with high sensitivity and specificity. The results confirm that the nanoarray selectively enriches tumor-associated EVs through EpCAM-aptamer interactions while preserving miRNA integrity, enabling precise detection of oncogenic biomarkers such as miRNA-21 and miRNA-23a. To evaluate the reusability of the gold nanoarray, the elution and regeneration cycle described in Methods section was repeated five times. SEM imaging [Figure S14A] confirmed that the nanoarray’s structural integrity was preserved after repeated use. After a single use, no notable residue remained, and the nanoarray maintained consistency with a freshly prepared substrate. After five cycles, minor surface residues, likely comprising residual EVs, fused vesicles, or exfoliated gold nanostructures, were observed and marked by yellow arrows in the SEM images. Additionally, the fluorescence signal after five reuse cycles retained approximately 90% of its original intensity [Figure S14B]. Statistical analysis indicated that fluorescence signal performance remained stable and statistically indistinguishable from the original for at least four cycles. These favorable reuse performances support the robustness and regenerative potential of the platform. The reusability can be attributed to the chemical and thermal stability of the gold nanoarray architecture. Collectively, these results support the feasibility of substrate regeneration and highlight the platform’s potential for cost-effective clinical translation. Building on this robust preclinical validation, we translated the platform to clinical sample analysis, utilizing plasma from CRC patients and healthy controls to assess its diagnostic potential. This progression underscores the platform’s translational utility in bridging in vitro models to real-world cancer diagnostics.

### Translating Fusion-Mediated EV miRNA Detection to Clinical Practice: Robust Performance in Colorectal Cancer Plasma Samples Using Gold Nanoarray and CRISPR/Cas13a

After validating the platform’s sensitivity and specificity using EVs from 2D cultures and a 3D VTS model, we translated the system to clinical plasma samples to evaluate its diagnostic potential for colorectal cancer. Plasma was collected from 5 CRC patients and 9 noncancerous controls, enabling a direct comparison of EV miRNA profiles between cancerous and noncancerous groups. This transition from preclinical models to human samples underscores the platform’s capacity to bridge in vitro validation with clinical utility. The histological and pathological details of the 14 human subjects are provided in Table S2. EVs were isolated from 250 μL of plasma for each subject and diluted 100-fold for NTA to determine size distribution and particle concentration [Figure S15 **and** Table S3]. The results showed no significant differences in the mean size or particle concentrations between the cancerous and noncancerous groups, indicating that traditional physical properties are insufficient for distinguishing these groups.

To evaluate the detection performance of the developed fusion assay on the gold nanoarray, experiments were conducted using EVs isolated from 50 μL plasma, with the final resuspended volume of isolated EVs adjusted to 100 μL in PBS. The fusion assay results were compared to different detection methods to assess overall assay performance. First, total RNAs were extracted from plasma EVs using TRIzol reagent, and CRISPR/Cas13a sensing probes were used to detect miRNAs directly in bulk solution. As shown in Figure 6A, this method did not yield statistically significant differences in miRNA-21 and miRNA-23a levels between the cancerous and noncancerous groups. The primary reason for the lack of sensitivity is attributed to RNA degradation and loss during the lysis and purification steps.^86,87^ The developed fusion-based method was then applied to detect target miRNAs in plasma EVs. As shown in Figure 6 **B (i)** **& (ii)** and Figure 6D, the fluorescence intensity of miRNA-21 and miRNA-23a was measured for each patient’s plasma EVs. Statistical analysis in Figure 6 **B (iii)** confirms that this approach effectively differentiates cancerous samples from noncancerous controls, demonstrating superior sensitivity and specificity compared to direct detection in bulk solution. The fusion-based method eliminates RNA degradation and confines the reaction to femtoliter-sized reactors, significantly increasing local signal intensity and enhancing detection efficiency. To validate these findings, RT-qPCR, the gold standard method, was used to analyze total RNAs extracted from plasma EVs. As shown in Figure 6C, RT-qPCR results confirmed that miRNA-21 and miRNA-23a levels were significantly higher in plasma EVs from CRC patients compared to those from noncancerous controls, validating the presence of these target miRNAs in cancer-derived EVs. While the results of RT-qPCR and the fusion-based method were comparable, the fusion assay on the gold nanoarray eliminates labor-intensive RNA preparation and amplification steps, offering a faster and more efficient alternative.

**Figure 6:**
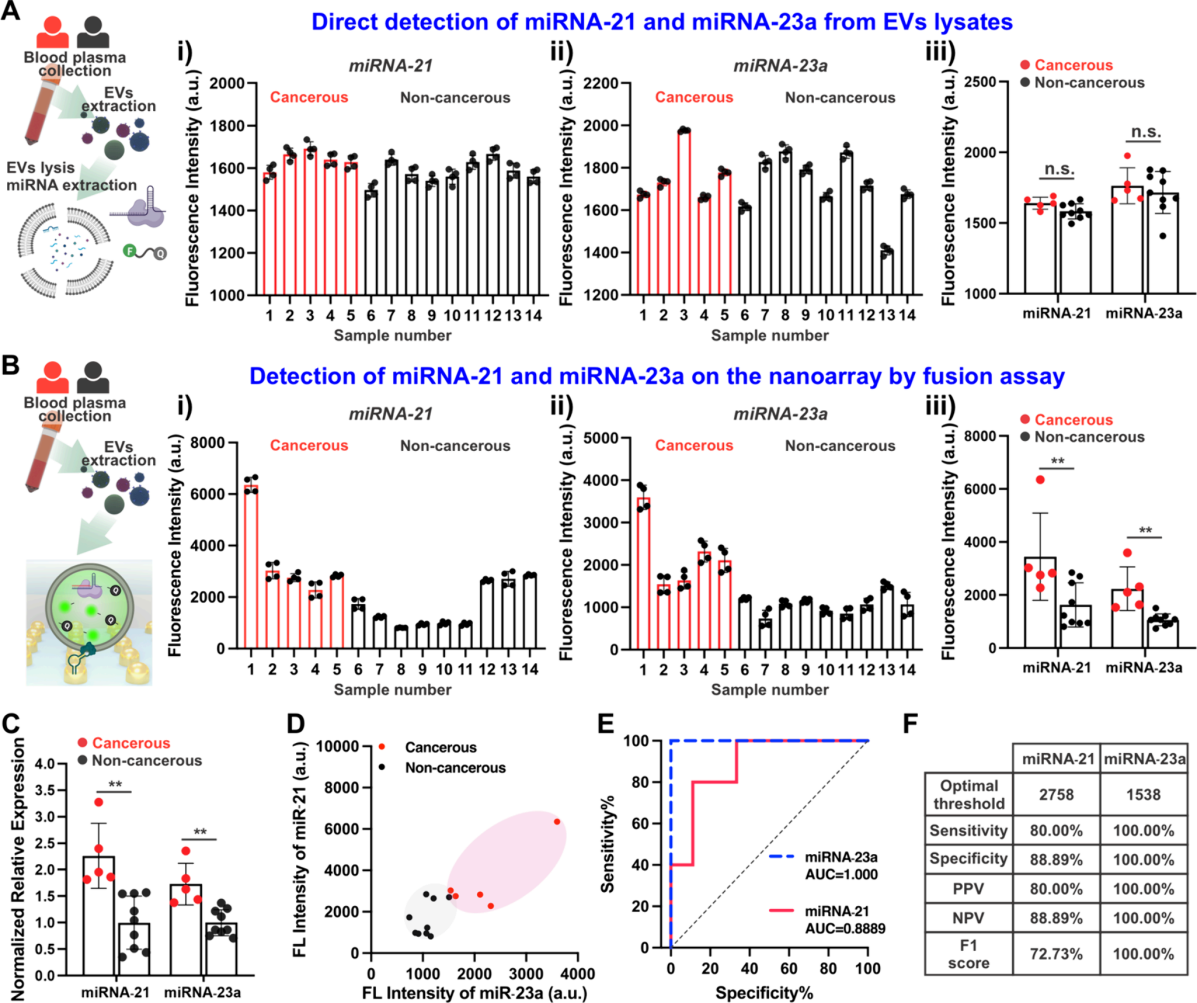
**Detection of clinical human plasma EV miRNAs.** (A) Direct detection of miRNAs using CRISPR/Cas13a sensing system from EV lysate in bulk. Fluorescent response of miRNA-21 (i) and miRNA-23a (ii) using method A (*n* = 4 replicates per sample, individual data point are plotted as dots, bar graph represents mean ± SD). (iii) Statistic analysis comparing target miRNAs of cancerous group and noncancerous group. Each data point represents the mean value of 4 replicates. (B) Fusion assay on gold nanoarray for target miRNAs detection. Fluorescent response of miRNA-21 (i) and miRNA-23a (ii) using method B (*n* = 4 replicates per sample, individual data point are plotted as dots, bar graph represents mean ± SD). (iii) Statistic analysis of target miRNAs of cancerous group and noncancerous group using fusion assay. Each data point represents the mean value of 4 replicates. (C) RT-qPCR assay as gold standard measurement for miRNAs detection. Statistic analysis of target miRNAs of cancerous group and noncancerous group using RT-qPCR (each data point represents the mean value of 4 replicates). (D) Fluorescence signal of miRNA-21 and miRNA-23a for 5 CRC patients and 9 noncancerous subjects using fusion assay on gold nanoarray. Gray circle represents noncancerous cases, while red circles represents cancerous cases. (E) ROC curves of miRNA-21 and miRNA-23a detection using fusion assay on gold nanoarray. (F) Statistic summary of ROC analysis. Statistic analysis was determined by two-tailed unpaired *t* test, n.s. *p* > 0.05, ** *p* < 0.01. Schematic created with BioRender.com.

To further assess the diagnostic utility of miRNA-21 and miRNA-23a, a receiver operating characteristic (ROC) analysis was conducted using the fusion-based method [Figure 6 **E** **& F**]. The area under the curve (AUC) for miRNA-23a was 1.000, indicating a nearly perfect ability to distinguish CRC patients from noncancerous controls with minimal false-positive rates. For miRNA-21, the AUC was 0.8889, demonstrating acceptable diagnostic performance. The lower AUC for miRNA-21 may be attributed to its role as a general marker for various diseases and inflammatory conditions, reducing its specificity for CRC. The inherent biological variability among patients can influence diagnostic accuracy, underscoring the limitations of relying solely on single biomarkers like miRNA-21 for CRC detection. Our findings demonstrate that combining miRNA-21 with miRNA-23a, two miRNAs with distinct but complementary roles in CRC progression, significantly enhances diagnostic robustness, mitigating the impact of individual variability. These results confirm that EV-derived miRNA-21 and miRNA-23a are synergistic biomarkers for liquid biopsy-based CRC diagnosis, offering a multiparametric approach to improve clinical decision-making. Furthermore, the gold nanoarray platform developed in this study demonstrates strong translational potential for clinical applications. By integrating high-specificity EV capture with CRISPR/Cas13a-activated miRNA sensing, the platform achieves sensitive, reproducible, and rapid detection of CRC biomarkers in complex biofluids. This clinic-ready technology addresses critical unmet needs in noninvasive cancer diagnostics, positioning it as a scalable solution for precision oncology.

## Conclusions

In this study, we designed and developed an integrated liquid biopsy platform meticulously engineered for the selective and highly sensitive detection of EV-associated miRNAs, with a primary focus on improving the early and accurate diagnosis of colorectal cancer. While EV-based diagnostics are actively explored for minimally invasive disease monitoring and management, conventional EV isolation techniques often lack the requisite specificity for precise downstream analysis. A critical challenge in the field is the selective isolation of tumor-derived EVs from the complex and heterogeneous milieu of biological fluids. This isolation is paramount for reliable biomarker discovery and subsequent analysis. Our platform directly addresses this challenge by synergistically integrating three technologies: (i) EpCAM-Specific Aptamer-Functionalized Gold Nanoarrays: these nanoarrays enable the highly selective capture of cancer-associated EVs, capitalizing on the overexpression of EpCAM on the surface of many tumor-derived EVs; (ii) CRISPR/Cas13a-Mediated miRNA Detection: this technology provides exceptional sensitivity and single-nucleotide specificity in detecting target miRNAs, surpassing the capabilities of traditional methods such as qPCR; and (iii) Liposome-Mediated Intracellular Delivery of CRISPR/Cas13a: this innovative delivery system facilitates the efficient transport of CRISPR/Cas13a components into the captured EVs, creating femtoliter-scale reaction chambers within intact EVs. This unique design significantly enhances signal amplification compared to conventional bulk EV analysis methods. By harnessing the combined power of these advanced biotechnologies, our platform represents an improvement toward developing more accurate, robust, and reliable EV-based diagnostic tools for early cancer detection and personalized monitoring.

This streamlined methodology circumvents labor-intensive and disruptive conventional workflows, such as EV lysis, RNA extraction, reverse transcription, and target amplification, by directly profiling miRNAs within intact EVs. This preservation of EV integrity minimizes RNA degradation and procedural variability, significantly enhancing detection efficiency. Integrating three core innovations, aptamer-mediated EV capture, CRISPR/Cas13a-based sensing, and liposome fusion-driven nanoreactors, the platform achieves a limit of detection of 2.5 × 10^1^ EV particles/μL, with a linear detection range from 10 to 10^6^ EV particles/μL, enabling precise quantification across clinically relevant EV concentrations. To comprehensively validate the platform’s performance, we tested it using EVs derived from three complementary sources: (i) 2D monoculture systems to establish baseline biomarker profiles, (ii) 3D VTSs, which closely replicate the complexity of the tumor microenvironment, and (iii) clinical plasma samples collected from CRC patients and noncancerous controls. This multitiered validation confirmed the platform’s robustness, reproducibility, and translational relevance. Furthermore, combining miRNA-21 and miRNA-23a as dual biomarkers improved diagnostic accuracy by mitigating individual variability, highlighting the value of multiplexed biomarker panels in reducing false positives/negatives. In addition, a comprehensive comparative summary of recently published EV miRNA detection methods is presented in Table S5. By integrating multiple nanobiotechnology, our platform stands out for its sensitive and selective detection of miRNAs, rapid assay turnaround, and thorough validation across advanced biological models.

While this study provides interesting evidence for the translational potential of EV miRNA profiling in clinical applications, particularly for colorectal cancer diagnosis, further investigation involving larger patient groups is warranted to rigorously establish the platform’s robustness and generalizability across diverse populations. To fully realize the capabilities of this liquid biopsy platform, future efforts will focus on expanding its multiplexing capabilities in two key areas. First, incorporating additional EV miRNA biomarkers will enhance the accuracy and reliability of diagnostic results. By developing comprehensive miRNA panels tailored to specific cancer types, including but not limited to colorectal cancer, we can improve the sensitivity and specificity of the diagnostic assay. Second, we aim to enhance the platform’s sensing performance to enable the interrogation of diverse EV contents at the single EV level. Achieving a single EV resolution will provide unprecedented insights into EV heterogeneity and the specific molecular signatures associated with different EV subpopulations. These advancements will not only deepen our understanding of the fundamental biological roles and functions of EVs but also expand their applications in theranostics. Ultimately, we envision this refined platform will support the flexibility of precision medicine, enabling more personalized and effective approaches to disease diagnosis, prognosis, and treatment.

## Methods

### Cell Culture

The human colorectal cancer cell line SW480 (ATCC) was cultured at a density of 1 million cells per 10 cm dish supplement with 10% (v/v) exosome-depleted FBS (Gibco, Cat# A2720803), penicillin (100 U/mL), and streptomycin (100 μg/mL) at 37 °C in a humidified incubator with 5% CO_2_ for 48 h. Primary Human Umbilical Vein Endothelial Cells (HUVECs, Lonza) were cultured in endothelial growth medium (EGM-2, Lonza) supplemented with 1% (w/v) penicillin/streptomycin (P/S, Gibco). Cells were maintained at passages below 6 to ensure viability and used in subsequent experiments. Normal human lung fibroblasts (LFs, Lonza) were cultured in fibroblast growth medium (FGM-2, Lonza) with 1% P/S and maintained up to Passage 6 to provide structural support within the vascularized spheroid.

### EV Isolation from Cell Culture Media Using the Optimized ATPS Method

EVs were isolated from cell-conditioned media using an aqueous two-phase system (ATPS), adapted from methods previously published.^78–80^ Conditioned media was subjected to a series of centrifugations at 4 °C to remove dead cells and large extracellular debris (500 g for 10 min, 2000 g for 10 min, followed by 10000 g for 30 min). Subsequently, polyethylene glycol (PEG) with a molecular weight of 20,000 and dextran with a molecular weight between 450,000 and 650,000 were dissolved in a solution of 200 mM Tris-HCl, 200 mM NaCl, and 10 mM Ethylenediaminetetraacetic acid (EDTA) at concentrations of 7.5% and 1.65% (w/v), respectively. This solution was added to the precentrifuged media in a 1:1 volume ratio and rotated overnight at 4 °C. The next day, the mixture was centrifuged at 1000 g for 10 min to remove the upper PEG phase, followed by further centrifugation at 3000 g for 10 min to pellet the EVs. The EV pellet was resuspended in DPBS and sterile filtered through a 0.22 μm membrane. This EV fraction was then characterized using a NanoSight NS300 instrument (Malvern Panalytical) to size distribution and number concentrations, and zeta potential and dynamic light scattering (DLS) measurements were performed using a ZetaSizer Nano NS (Malvern Panalytical).

### Plasma EV Isolation

EVs were isolated from plasma samples using an ExoQuick ULTRA EV Isolation Kit (EQULTRA-20A-1, System Biosciences) following the manufacturer’s instructions. Briefly, 250 μL plasma aliquots were centrifuged at 3,000g for 15 min to remove cell debris. The supernatants were then gently mixed with 67 μL ExoQuick solution from the kit and incubated on ice for 30 min. The mixture was then centrifuged at 3,000 g at 4 °C for 10 min. The resulting EV pellets were further processed following the manufacturer’s protocol.

### Size Distribution, Particle Concentration, and Zeta Potential Measurements of EVs and Liposomes

The size distributions and particle concentrations of EVs and liposome samples were determined using a NanoSight NS300 instrument (Malvern Panalytical) and analyzed by the Nanoparticle Tracking Analysis (NTA) 3.4 Software (Malvern Panalytical). Each sample was measured five times with a capture duration of 60 s per measurement, following the standard measurement protocol. Zeta potential and dynamic light scattering (DLS) analyses were performed using a ZetaSizer Nano NS (Malvern Panalytical) to further characterize the samples.

### TEM Analysis

The EV, liposome and fused vesicles were prepared to a final concentration of 1 x10^6^ vesicles per μL in PBS. A 10 μL aliquot of each sample was deposited onto a carbon-coated grid, and excess liquid was removed using Kimwipes. The samples were then negatively stained with 5 μL 2% uranyl acetate for 1 min. Excess stain was wicked away with Kimwipes, and the grids were allowed to dry completely under vacuum at room temperature. Images of EVs, liposomes, and fused vesicles were captured at 80 kV using a Philips CM12 transmission electron microscope (TEM) equipped with an AMT digital camera (model: XR111).

### Field Emission-Scanning Electron Microscopy (FE-SEM) Analysis

Tumor spheroid samples were washed three times with DPBS and fixed using 4% formaldehyde on glass in DPBS for 10 min at RT. The fixed cells were subjected to graded dehydration using various concentrations of ethanol (30%, 50%, 70%, 90%, and 100%, each for 10 min) and hexamethyldisilazane (HMDS) (50% and 100%, each for 10 min). The dehydrated cells were vacuum-dried overnight. Afterward, the glass with the dried spheroids was mounted onto stubs and coated with 15 nm of gold using a sputter coater. The morphology of the tumor spheroid was then observed using FE-SEM (10 kV, Carl Zeiss, Germany). The images obtained from FE-SEM were pseudo colored using Adobe Photoshop.

### Atomic Force Microscopy Analysis

The gold nanoarray was characterized using an atomic force microscope (AFM, Park Systems, NX10) both before and after the capture of EVs. Noncontact mode was employed to image the surface of each substrate at the nanometer scale. Surface height profiles and 3D images were obtained and analyzed using the XEI software program.

### Western Blot

EV markers and other protein contents were analyzed using Western blotting. Cells and EVs were lysed in RIPA lysis buffer supplemented with a protease inhibitor and incubated on ice for 30 min. Proteins were denatured at 95 °C for 10 min after adding 5× sample loading buffer. The samples were then separated on an SDS-PAGE gel, with electrophoresis conducted at 80 V for 30 min followed by 100 V for 60 min. The separated proteins were transferred onto a 0.45 μm nitrocellulose membrane (Invitrogen, Cat# STM2008) at 100 V for 40 min.

The membranes were blocked with 5% BSA at room temperature for 1 h and subsequently incubated with primary antibodies overnight at 4 °C. The primary antibodies and their dilutions used included β-actin (Cell Signaling Technology, CST, Cat# 3700S, 1:1000), CD81 (CST, Cat# 56039S, 1:1000), CD63 (BioLegend, Cat# 353003, 1:500), and EpCAM (CST, Cat# 2929s, 1:1000). After incubation, the membranes were washed three times with Tris-buffered saline containing 0.05% Tween 20 (TBS-T), with each wash for 5 min. HRP-linked secondary antibodies, antimouse IgG (CST, Cat# 7076S, 1:1000), and antirabbit IgG (CST, Cat# 7074S, 1:1000), were applied to the membranes for 1 h at room temperature. The washing steps with TBS-T were repeated three times. Immunoblots were visualized using an enhanced chemiluminescence (ECL) substrate (Thermo Fisher Scientific, Cat# 34580) and imaged using the iBright FL1000 imaging system (Thermo Fisher Scientific).

### RT-qPCR Assay

RT-qPCR was used to quantify miRNAs expression level. EV miRNAs were extracted using TRIzol reagent (Invitrogen, Cat# 15596026) based on the manufacturer’s instructions. The expression level of miRNA 23a and miRNA 21 was quantified using the miRCURY LNA miRNA PCR Starter Kit (QIAGEN, Cat# 339320). qRT-PCR was performed on the StepOnePlus real-time PCR system (Applied Biosystems). miRNA 103 served as an internal control. Relative expression levels were calculated using the 2−ΔΔCt method.

### CRISPR/Cas13a-Mediated Trans-cleavage Assay

The CRISPR/Cas13a-mediated trans-cleavage assay, also referred to as collateral activity, was conducted in 1× Cas13a reaction buffer consisting of 20 mM HEPES, 100 mM NaCl, 5 mM MgCl_2_, and 10 μM EDTA (pH 6.8). Each 100 μL reaction contained 50 nM purified LwaCas13a (Molecular Cloning Laboratories, Cat# CAS13a-100), 50 nM crRNA (Integrated DNA Technologies, USA), 250 nM quenched fluorescent RNA reporter (Integrated DNA Technologies, USA), 1 U/mL murine RNase inhibitor (New England Biolabs), and varying concentrations of RNA targets. LwaCas13a and crRNA were mixed and incubated at room temperature for 15 min to form the Cas13a/crRNA ribonucleoprotein (RNP) complex. Subsequently, RNase inhibitor, RNA reporter, and the RNA targets were added to the reaction mixture. The assay was incubated at 37 °C, and fluorescence intensity was measured at designated time intervals using a TECAN plate reader. The excitation wavelength was set to Ex = 488 nm, and emission was recorded at Em = 522 nm.

### Synthesis of Liposome Particles and CRISPR/Cas13a RNP-Encapsulated Liposomes

Liposome particles and CRISPR/Cas13a ribonucleoprotein (RNP)-encapsulated liposomes were synthesized using the thin-film hydration and extrusion method. Lipids used in this study included 1,2-dioleoyl-*sn*-glycero-3-phosphoethanolamine (DOPE) and 1,2-dioleoyl-3-trimethylammonium-propane (DOTAP), both prepared as 20 mM stock solutions in chloroform. Additional lipids and, including 1,2-distearoyl-*sn*-glycero-3-phosphoethanolamine-N [maleimide (polyethylene glycol)-2000] (DSPE-PEG-2000), Lissamine Rhodamine B 1,2-dihexadecanoyl-*sn*-glycero-3-phosphoethanolamine, triethylammonium salt (Rhod B DHPE), 23-(dipyrrometheneboron difluoride)-24-norcholesterol (TopFluor Cholesterol) and cholesterol, were reconstituted at a concentration of 1 mg/mL. Lipids were purchased from Avanti. All stock solutions were stored at – 20 °C until use. Lipids were mixed in 1 mL chloroform in a glass vial to form a mixture consisting of 50 μM DOPE, 30 μM DOTAP, 5 μM DSPE-PEG-2000, and 20 μM cholesterol. For the FRET assay, a FRET pair comprising 1 μM TopFluor cholesterol and 1 μM Rhod B DHPE was included in the mixture, along with 48 μM DOPE or DMPC, 30 μM DOTAP, 5 μM DSPE-PEG-2000, and 15 μM cholesterol. The detailed lipid compositions are provided in Table S1. The lipid mixture was dried under argon to create a homogeneous thin lipid film on the inner surface of a glass vial. The dried film was hydrated by adding 200 μL HEPES buffer (pH 7.4) or 200 μL of 50 nM CRISPR/Cas13a RNP, followed by sonication for 30 min to achieve uniform hydration. The resulting suspension was extruded through 100 nm polycarbonate membranes using an Avanti Mini Extruder to generate uniform liposomes and CRISPR/Cas13a RNP-encapsulated liposomes. Free reagents and excess lipids were removed using a size-exclusion chromatographic resin column. The final liposome solution, with a concentration of 2 × 10^9^ liposomes/mL, was aliquoted and stored at 4 °C. Aliquots were diluted as necessary for subsequent fusion assays and FRET assays.

### FRET Assay and Fusion Assay in Solution

The fusion of EVs with FRET pair-doped liposomes was assessed using a fluorescence resonance energy transfer (FRET) assay. EVs and liposomes were prepared at a concentration of 1 × 10^9^ particles/mL for each and incubated together at 37 °C for 1 h. Fluorescence signals were excited at 488 nm, and emission spectra were recorded from 450 to 650 nm using a fluorescence spectrophotometer (Agilent Technologies). The fusion assay between EVs and liposomes in bulk solution was conducted as described above.

### Detection of EV miRNAs on Gold Nanoarray

For the detection of EVs miRNAs on a gold nanoarray, 100 μL EVs isolated from cell-conditioned media at varying concentrations or 100 μL EVs isolated from clinical plasma were added to the EpCAM aptamer modified gold nanoarray substrate and incubated at room temperature for 1 h. Uncaptured EVs were removed by washing the substrate with PBS. Subsequently, 100 μL of Cas13a/crRNA RNP-encapsulated liposomes (2 × 10^9^ particles/mL) were added to the substrate and incubated at 37 °C for 1 h. Fluorescence intensity was measured using a TECAN plate reader.

### Reuse Performance Evaluation

To assess the reusability of the gold nanoarray, a heat elution strategy was employed.^88–90^ After completing a single fusion assay, the nanoarray was placed on a metal heating block at 95 °C for 10 min to thermally dissociate the surface-bound EVs and aptamers. Following the initial elution, the surface was thoroughly rinsed, and this thermal treatment was repeated twice to ensure complete removal of residual biological components. The cleaned nanoarray surface was then refunctionalized with freshly prepared EpCAM aptamers and subjected to a new cycle of EV capture and fusion assay.

### Hydrogel Microwell Fabrication

Hydrogel microwell arrays were fabricated as described in previous studies.^85^ Briefly, 9 mm × 9 mm glass slides were used as substrates to support the hydrogel microwell arrays. The slides were treated with 3-(trimethoxysilyl) propyl methacrylate (TMSPMA, Sigma-Aldrich, USA) and heated at 65 °C overnight to enhance adhesion to the hydrogel. A 15 μL aqueous solution containing 10% (w/w) PEG 1,000 dimethacrylate and 1% (w/w) of the photoinitiator 2-hydroxy-4’-(2-hydroxyethoxy)-2-methylpropiophenone (Sigma-Aldrich, USA) was applied between the glass slide and a PDMS stamp. Hydrogel polymerization was initiated via a radical chain growth reaction under UV light (320–350 nm) for 30 s. After polymerization, the PDMS mold was carefully peeled away from the glass slide, and the hydrogel microwell arrays were washed with ethanol. The arrays were then stored in PBS overnight before use.

### SW480 Tumor Spheroid Culture on Hydrogel Microwells

A total of 900,000 SW480 cells and 300,000 lung fibroblast cells were mixed in 500 μL of SW480 media supplemented with 5% Matrigel as an extracellular matrix. The cell mixture was seeded into hydrogel microwell arrays and incubated overnight at 37 °C in a cell incubator (Day −1). Tumor spheroids formed the following day (Day 0) and were subsequently used for downstream experiments.

### Fabrication of Vascularized Tumor Spheroids on a Chip

On Day 0, the Sphero-IMPACT chip was prepared by attaching a silicone adhesive film (IS-00820, IS Solutions) to the bottom of the chip and exposing it to O_2_ plasma (Femto) for 1 min. A vascularization cell mixture containing 12 million cells/mL human umbilical vein endothelial cells (HUVECs), 8 million cells/mL lung fibroblasts, 2.5 mg/mL fibronectin, 0.5 U/mL thrombin, and 0.15 U/mL aprotinin was prepared. A total of 7 μL of this mixture was injected into each chamber of the chip, along with one SW480 tumor spheroid. To support vascular network formation, 200 μL of EGM-2 media was added to each chamber. The cells were cultured in the Sphero-IMPACT chip for 7 days, with media being refreshed daily. Over this period, vascularization was observed, resulting in the formation of an intricate, vascularized 3D tumor spheroid model. The cell-conditioned media was collected for EV isolation and analysis.

### Collection of Clinical Samples

Human whole blood samples were collected in EDTA-coated blood collection tubes from five colorectal cancer patients and nine noncancerous controls. The plasma samples were isolated and processed from whole blood immediately upon whole blood collection by the Rutgers Cancer Institute of New Jersey (CINJ) Biospecimen Repository and Histopathology Service Shared Resource. The study was approved by the Institutional Review Board (IRB) of the Rutgers University Human Research Protection Program (HRPP) under IRB Study ID: Pro2023000414. The obtained plasma samples were aliquoted in 1 mL/vial and stored at liquid nitrogen tank for preservation.

### Materials for LIL

ITO electrodes were purchased from MSE Supplies, USA. All the materials used for laser interference lithography (LIL) process including photoresist (AZ2020), solvent (AZ EBR Solvent), developer (AZ MIF3000), and stripper (AZ 400T) were obtained from AZ Electronic Materials, USA. The SYLGARD 184 silicon elastomer base and curing agent (Polydimethylsiloxane, PDMS) was purchased from Dow Corning, USA. The Au plating solution was purchased from Alfa Aesar, USA.

### Fabrication of PR Nanoparticle Hole Array

First, indium tin oxide (ITO)-coated glass (15 Ω/cm^2^, 0.7 mm thickness, 10 mm × 10 mm, MSE supplies) was sonicated in 1% Triton X-100, deionized water, and 70% ethanol for 20 min each to clean the ITO substrates. The ITO substrates were stored in 70% ethanol, and dried using N_2_ gas before pattern fabrication. To fabricate the nanoparticle hole array, a photosensitive layer known as the photoresist (AZ2020) diluted with its solvent (AZ EBR solvent) with a ratio of 3:2 was spin-coated (Laurell Technologies, USA) onto a cleaned ITO surface. Next, substrates were prebaked on a hot plate for 60 s at 125 °C. Afterward, the substrate was exposed to the UV light (λ = 325 nm, 0.81 mW) by the light source (He–Cd laser, Kimmon Koha Laser Systems, Japan) with a Lloyd’s mirror interferometer (Thor Laboratories) for 16 s. The simultaneous reflected light coming from the Lloyd’s mirror interferometer results in a periodic interference pattern that cross-links exposed areas of the PR creating a regular PR pattern on the substrate. The angle of the sample holder incorporating the Lloyd’s mirror interferometer was adjusted to generate nanoparticle hole arrays with different sizes according to the equation given by

Λ=λ/2sinθ

Where Λ is the size of the pitch (nm), λ is the wavelength of the UV laser (325 nm), and θ is the incident angle (°). The incident angle of laser exposure (8°) resulted in a 1200 nm pitch size. To create the nanoparticle hole array, the ITO substrate was rotated 90° relative to its position for the first exposure and exposed to the LIL UV light again for 16 s. Immediately afterward, the substrate was postbaked on a hot plate for 60 s at 125 °C. The unexposed photoresist was removed by immersing the substrate into the developer solution (AZ 300 MIF developer) for 32 s followed by washing with DI water. Finally, a periodic nanoparticle hole array was generated on the ITO substrate. Substrates were subjected to oxygen plasma (Femto Science Inc., Korea) treatment to remove residual PR on the ITO surface (140W, 50 sccm of O_2_) for 2 min and further baked on a hot plate for 60 s at125 °C prior to electrochemical gold deposition.

### Fabrication of Gold Nanostructured Array on ITO Substrate

First a cylindrical plastic chamber was attached onto the patterned ITO substrate using PDMS with a ratio of 10:1 between PDMS and thermal curing agent. Next, a three-electrode system was constructed consisting of a silver/silver chloride (Ag/AgCl) reference electrode, a platinum wire counter electrode, and the ITO substrate as the working electrode. Afterward, Au plating solution (Alfa Aesar, USA) was pipetted into the small chamber. DC amperometry (CHI 600E electrochemical workstation, CH Instruments, Inc., USA) was performed at a potential of −1.2 V for 24 s to electrochemically deposit gold into the nanoparticle hole array PR pattern. Finally, the remaining PR on the substrate was removed by soaking the substrate in the stripper solution (AZ 400T Stripper) for 70 min at 65 °C followed by washing with 70% ethanol and DI water.

### Statistical Analysis

Data generated from the study were analyzed using GraphPad Prism 10 software (GraphPad). Multiple group comparisons (group >2) were performed using one-way ANOVA, while two-group comparisons were conducted using a two-tailed unpaired *t* test. Statistical significance was defined as n.s. *p* > 0.05, ** *p* < 0.01, **** *p* < 0.0001.

## Supplementary Material



## Notes

Schematic description of EV isolation, including gel electrophoresis, optimization of EV isolation, DLS analysis, zeta potential, confocal images of vascularization, TEM of photoresist, electrochemical properties of nanoarray, lipid composition of liposomes, oligos sequence, patients’ information and comparative summary (PDF)
