# Supplementary material for: Advanced Cancer Liquid Biopsy Platform for miRNA Detection in Extracellular Vesicles Using CRISPR/Cas13a and Gold Nanoarrays
Source: ACS Nano. 2025 Jul 28;19(35):31438–56. doi: 10.1021/acsnano.5c06940 (PMC12424298; doi:10.1021/acsnano.5c06940)
Supplement: Supplementary file 1 [file nn5c06940_si_001.pdf]

SUPPORTING INFORMATION

Advanced Cancer Liquid Biopsy Platform for miRNA Detection in Extracellular Vesicles Using CRISPR/Cas13a and Gold Nanoarrays

Meizi Chen[1], Hye Kyu Choi,[1,2], Li Ling Goldston[1], Yannan Hou[1], Caiping Jiang[1] and Ki-Bum Lee[1]*

[1]Department of Chemistry and Chemical Biology, Rutgers, The State University of New Jersey,123 Bevier Road, Piscataway, NJ 08854, USA

[2]Department of Chemical and Biomolecular Engineering, Sogang University, Seoul, 04107, Republic of Korea

CORRESPONDING AUTHOR:

KIBUM LEE

DEPARTMENT OF CHEMISTRY AND CHEMICAL BIOLOGY, RUTGERS, THE STATE UNIVERSITY OF NEW JERSEY

TEL. +1-732-445-2081; FAX: +1-732-445-5312

EMAIL: kblee@rutgers.edu

https://kblee.rutgers.edu/

KEYWORDS: Nano liquid biopsy, Extracellular vesicle (EV) biomarkers, MicroRNA detection, Aptamer-functionalized nanoarrays, CRISPR/Cas13a diagnostics, Nanoscale reactors, Vascularized tumor spheroid, Non-invasive cancer detection

TABLE OF CONTENTS

Figure S1-- 3

Figure S2-- 4

Figure S3-- 5

Figure S4-- 6

Figure S5-- 7

Figure S6--8

Figure S7-- 9

Figure S8--- -10

Figure S9--- 11

Figure S10--12

Figure S11--13

Figure S12--14

Figure S13--15

Figure S14--16

Figure S15--17

Table S1---18

Table S2---19

Table S3---20

Table S4---21

Table S5---22

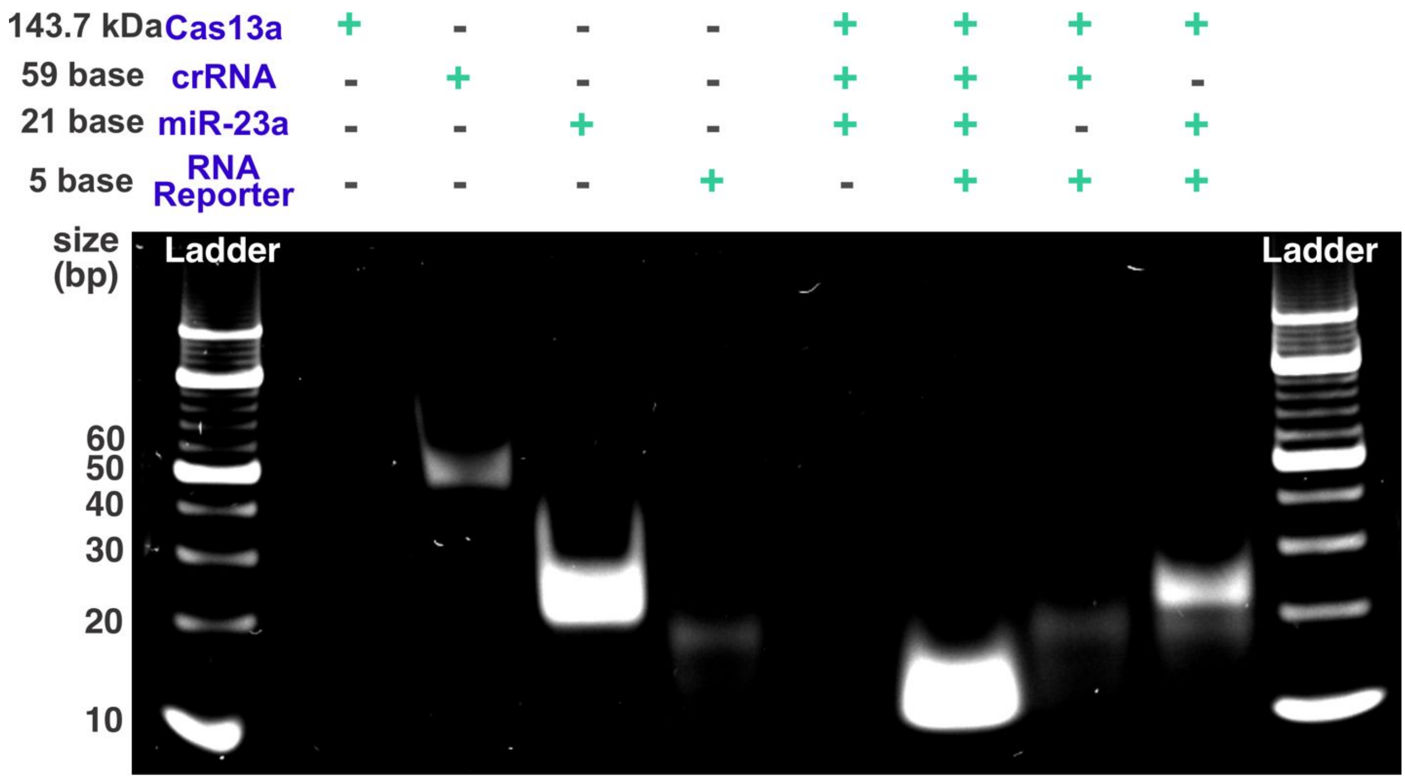

Figure S1. Gel electrophoresis analysis of CRISPR/Cas13a trans-cleavage activity. Gel electrophoresis results demonstrate that trans-cleavage by the activated CRISPR/Cas13a complex occurs only in the presence of all required components: Cas13a enzyme, crRNA, RNA target, and RNA reporter.

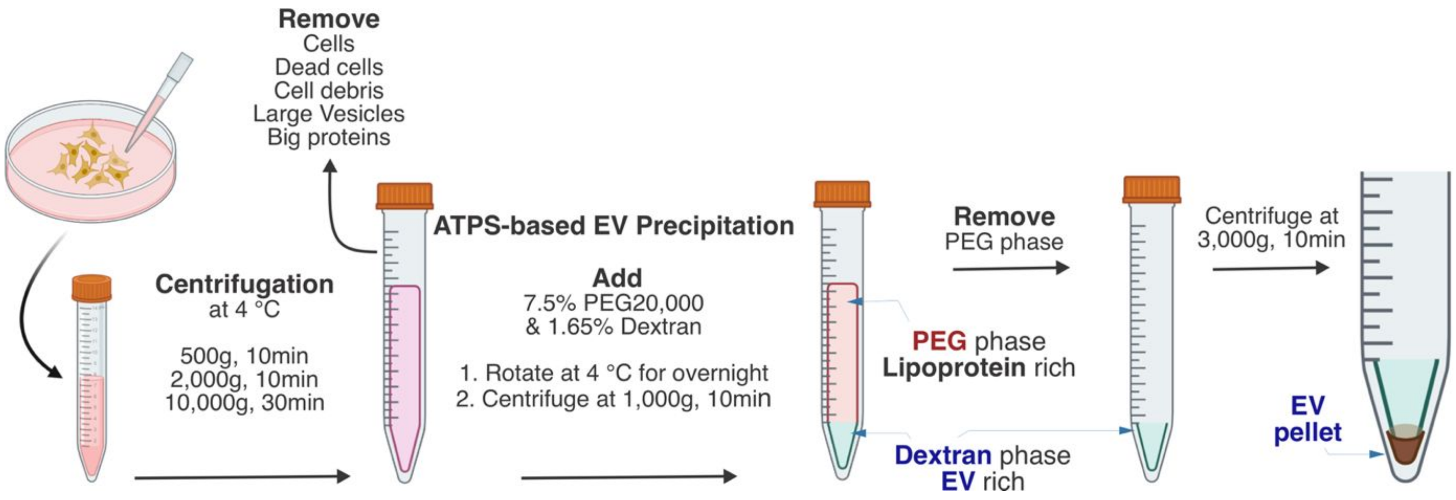

Figure S2. Optimized aqueous two-phase system (ATPS) method for EV isolation from cell-conditioned media. Schematic representation and experimental flow of the optimized ATPS method for efficient EV isolation from cell-conditioned media.Schematic created with BioRender.com.

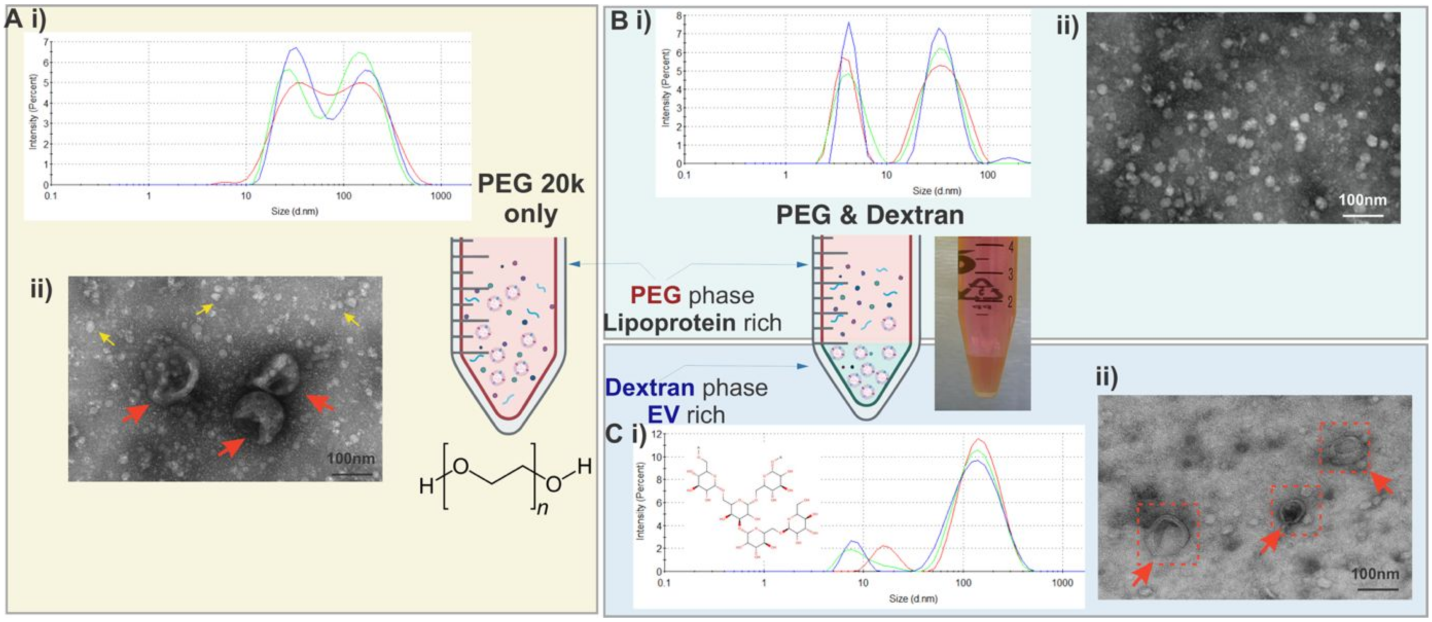

Figure S3. Comparison of the PEG-only EV precipitation method with the ATPS method. (A) i) DLS analysis of EVs isolated using the PEG-only precipitation method. ii) TEM images of EVs obtained from the PEG-only method. Yellow arrows indicate lipoproteins, while red arrows highlight EVs. (B) i) DLS data showing size distribution and (ii) TEM images of the PEG phase from the ATPS method. (C) i) DLS data showing size distribution and (ii) TEM images of the dextran phase from the ATPS method, demonstrating that the ATPS method selectively enriches EVs in the dextran phase, while the PEG phase primarily retains lipoproteins. Red arrows and a dotted box highlight EVs. Schematic created with BioRender.com.

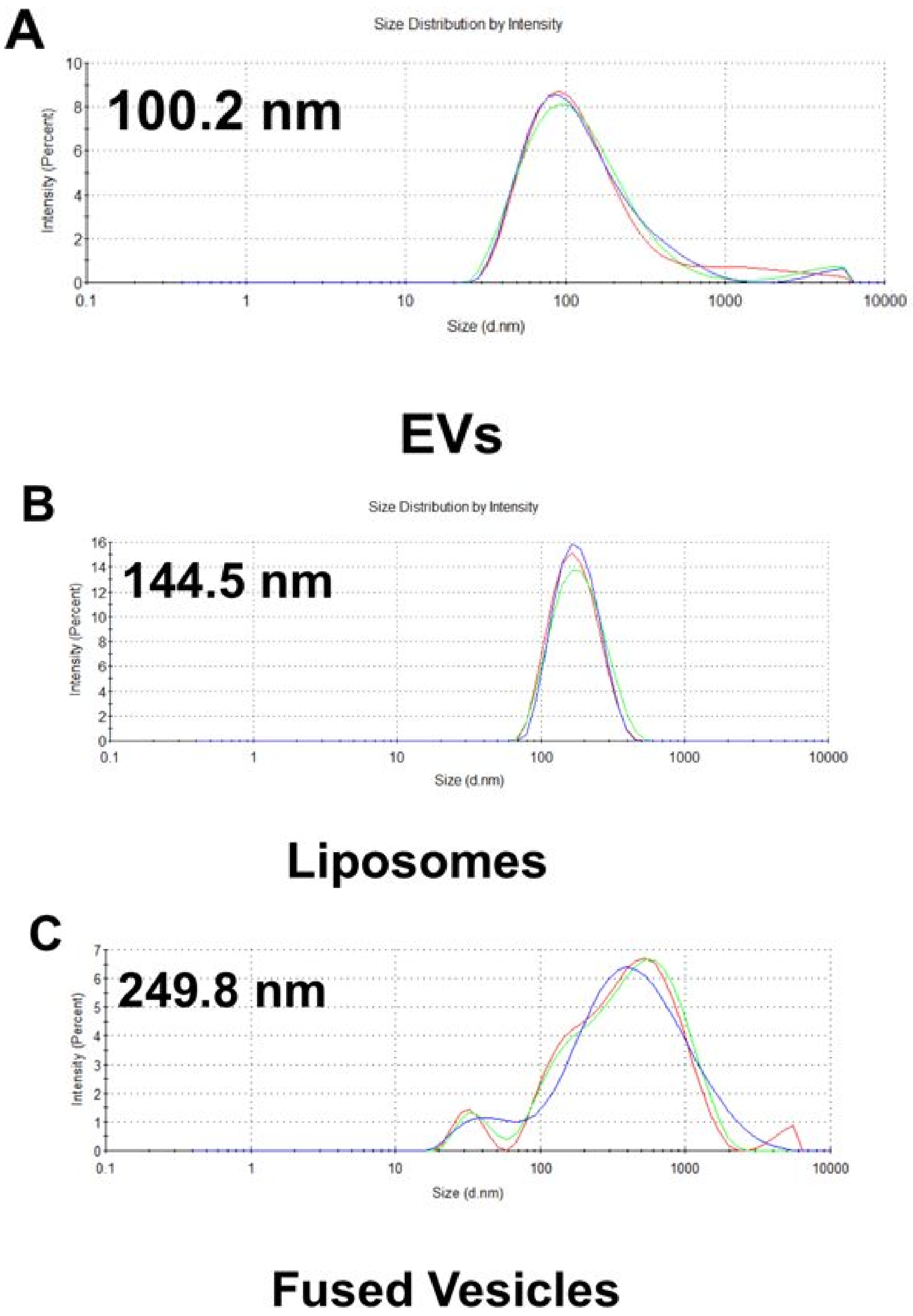

Figure S4. DLS analysis of EVs, liposomes, and fused vesicles. DLS measurements displaying the size distribution profiles of EVs (A), synthetic liposomes (B), and fused vesicles (C), with peak size values labeled for each respective measurement.

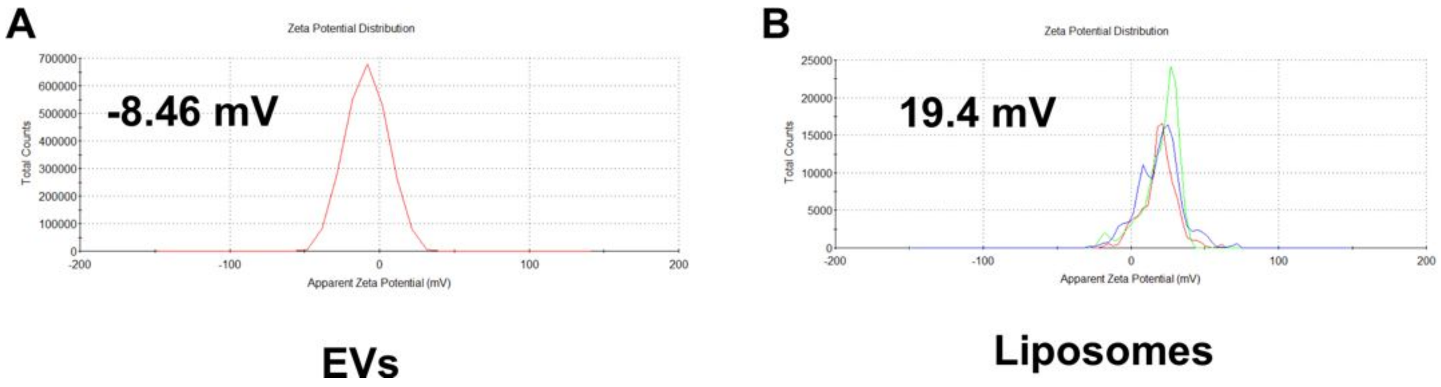

Figure S5. Zeta potential analysis of EVs and liposomes. Zeta potential measurements of EVs (A) and synthetic liposomes (B), with peak zeta potential value labeled.

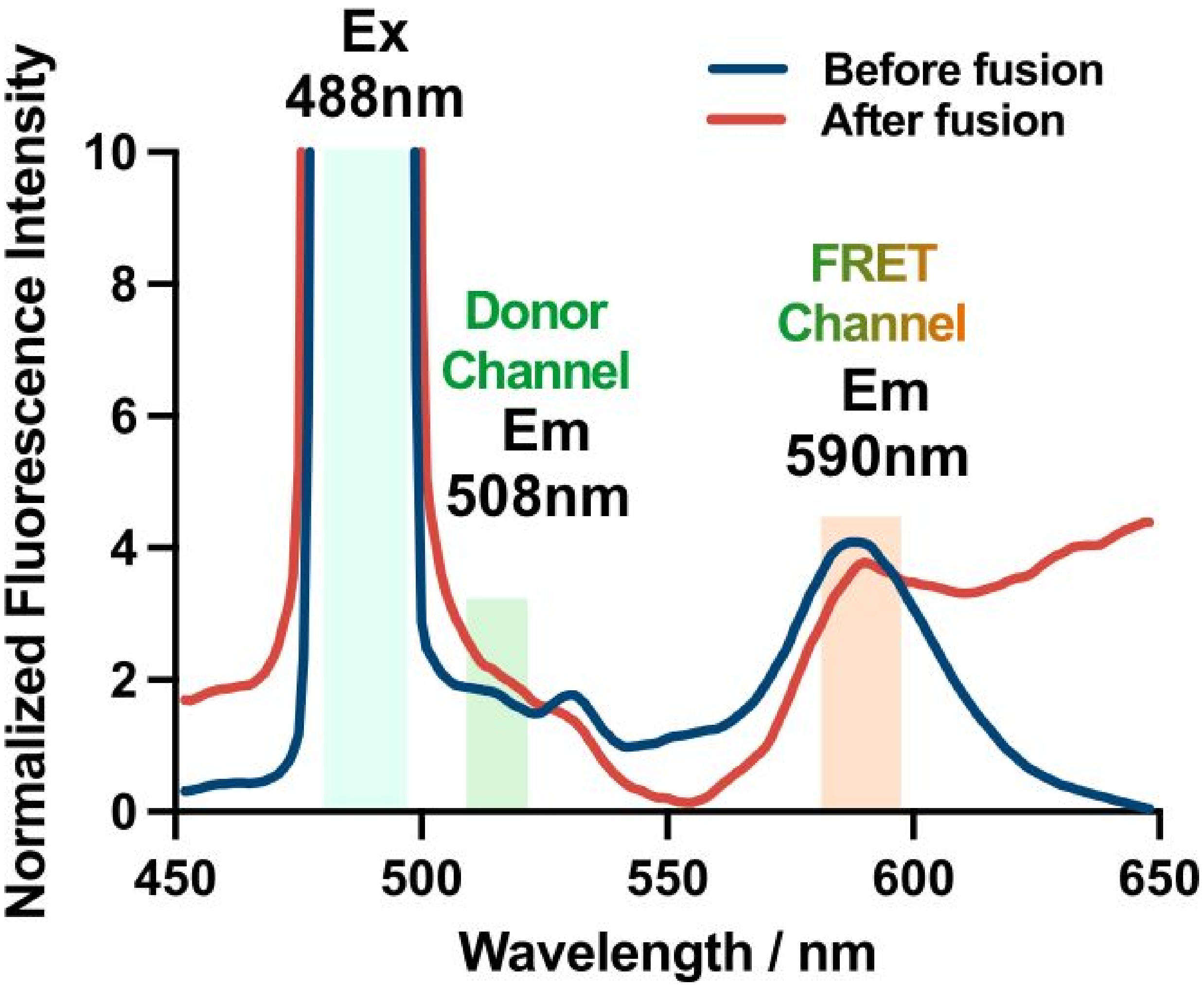

Figure S6. FRET emission profiles of non-fusogenic liposomes and EVs. FRET emission results demonstrate a reduced spectral change compared to those observed with fusogenic liposomes, indicating that liposomes lacking fusogenic lipid components are unable to effectively fuse with EV membranes.

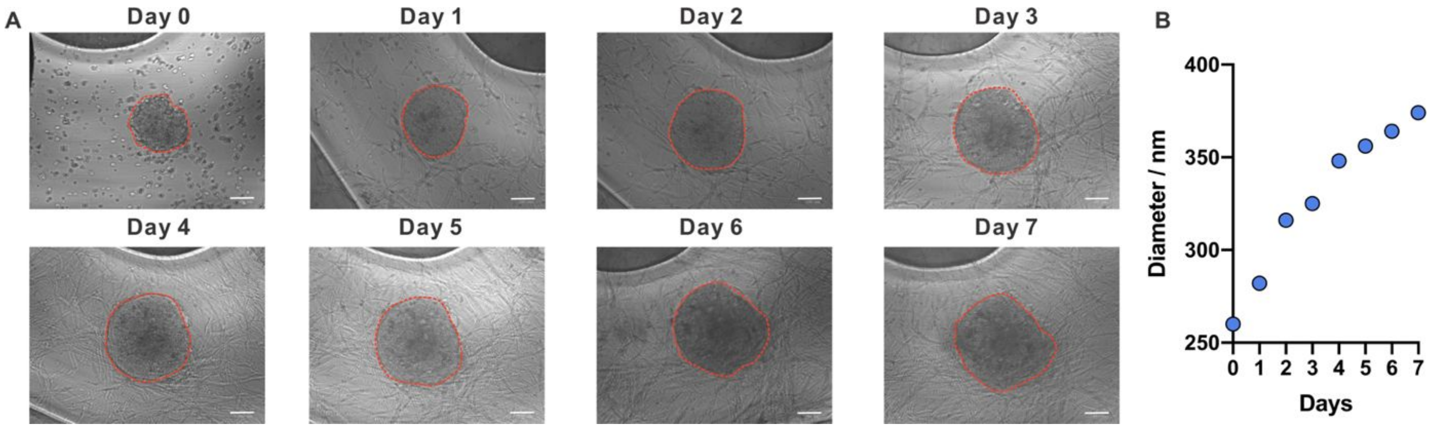

Figure S7. Tumor spheroid seeding and vascular structure formation in a microchip over time. (A) Brightfield images showing the progression of tumor spheroid growth and vascular structure formation in the microchip from day 0 to day 7. The red dotted line outlines the tumor spheroid. (B) Quantitative analysis of tumor spheroid diameter over the 7-day growth period in the microchip. Scale bars: 100 μm.

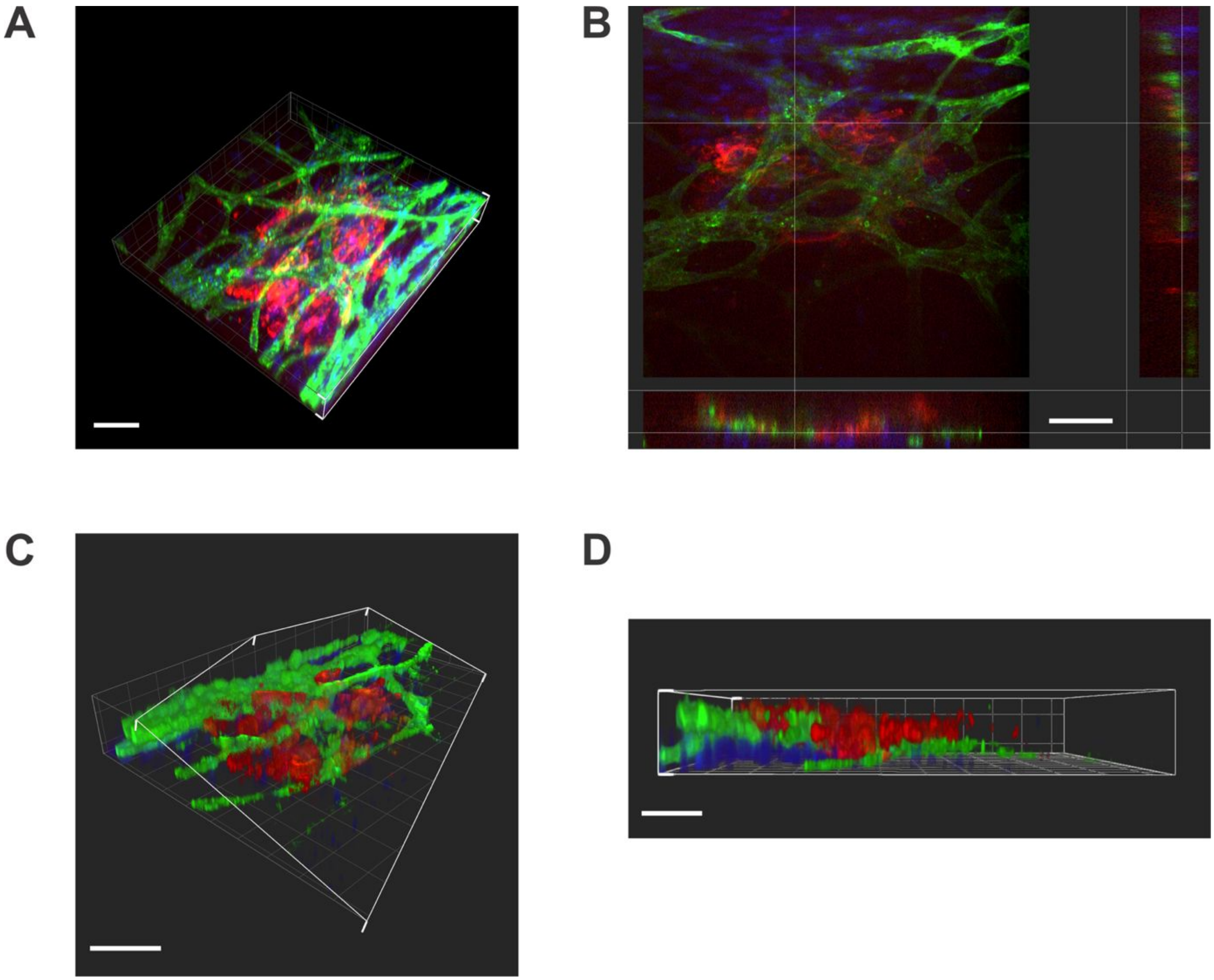

Figure S8. 3D confocal imaging of a vascularized tumor spheroid in a microchip. (A) 3D confocal image showing the interaction between the vascular network (Lectin, green) and the tumor spheroid (EpCAM, red) within a 3D microenvironment. Nuclei are stained in blue. (B) Representative single-plane confocal image illustrating the spatial organization of the vascular network (Lectin, green) and tumor spheroid (EpCAM, red) in the 3D microenvironment. The orthogonal views on the right and bottom panels provide cross-sectional perspectives, confirming the integration of vascular structures with the tumor spheroid. Nuclei are stained in blue. (C) 3D projection image highlighting the depth of the vascular network surrounding the tumor spheroid, as well as vascular structures within the spheroid lumen. (D) Side view cross-sectional 3D confocal image demonstrating the spatial distribution of vascular and tumor structures. Nuclei are stained in blue. Scale bars: 100 µm.

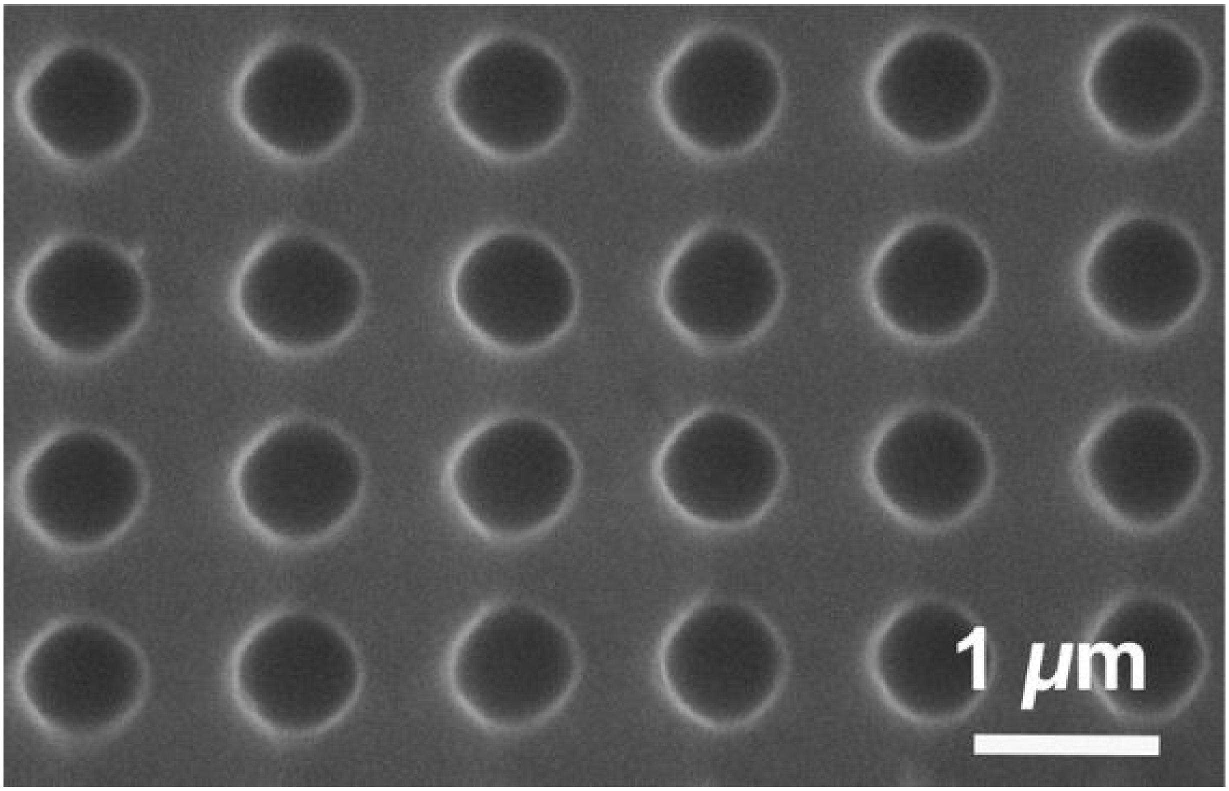

Figure S9. SEM image of photoresist pattern after laser interference lithography (LIL) treatment. SEM image of the photoresist pattern following LIL treatment, illustrating the structural morphology and pattern uniformity achieved through the fabrication process. Scale bars: 1 μm.

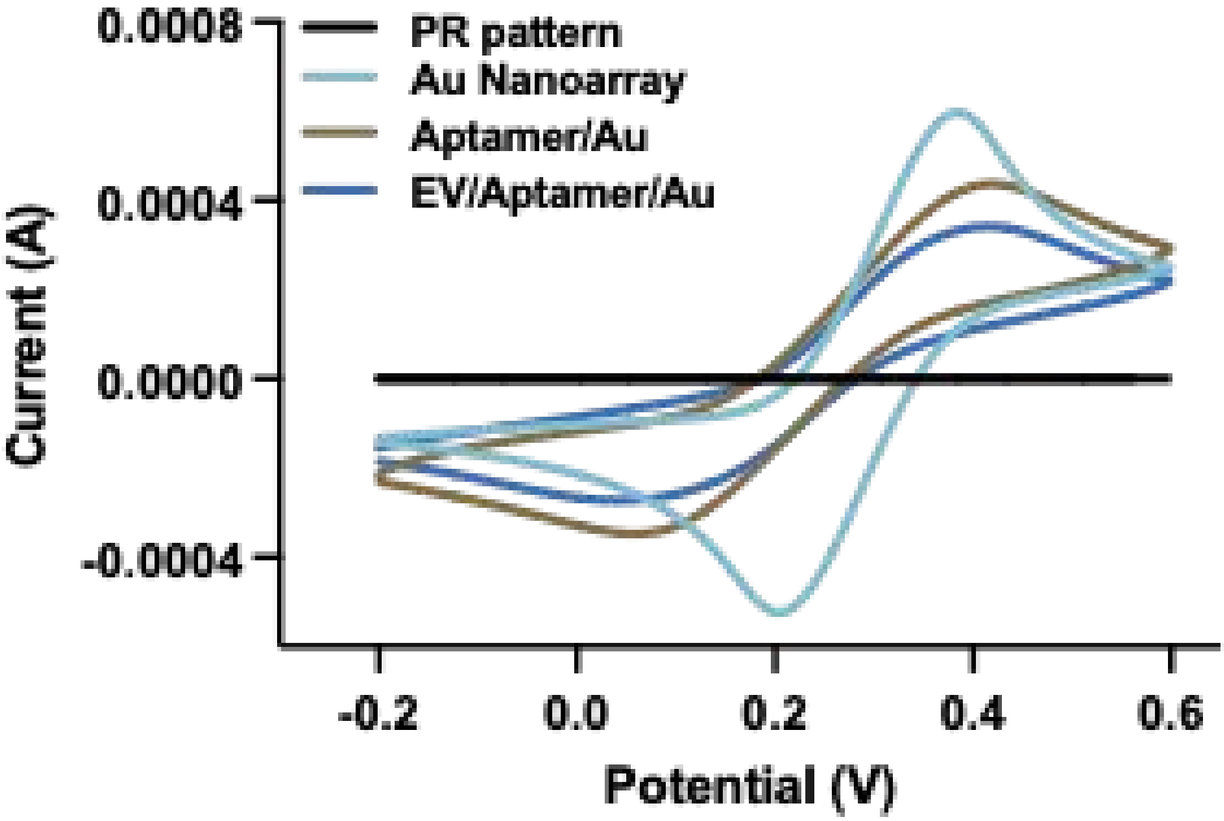

Figure S10. Cyclic voltammetry analysis of different gold nanoarray modifications. Cyclic voltammetry (CV) curves of the photoresist pattern, Au nanoarray, EpCAM aptamer-functionalized Au nanoarray, and EV-captured gold nanoarray (EV/aptamer/Au) recorded in 0.1 mol L^{-1} PBS (pH 7.40) containing 5 mmol L^{-1} $[Fe(CN)_6]^{3-/4-}$ and 0.1 mol L^{-1} KCl. The electrochemical response demonstrates the stepwise functionalization and EV capture on the gold nanoarray surface.

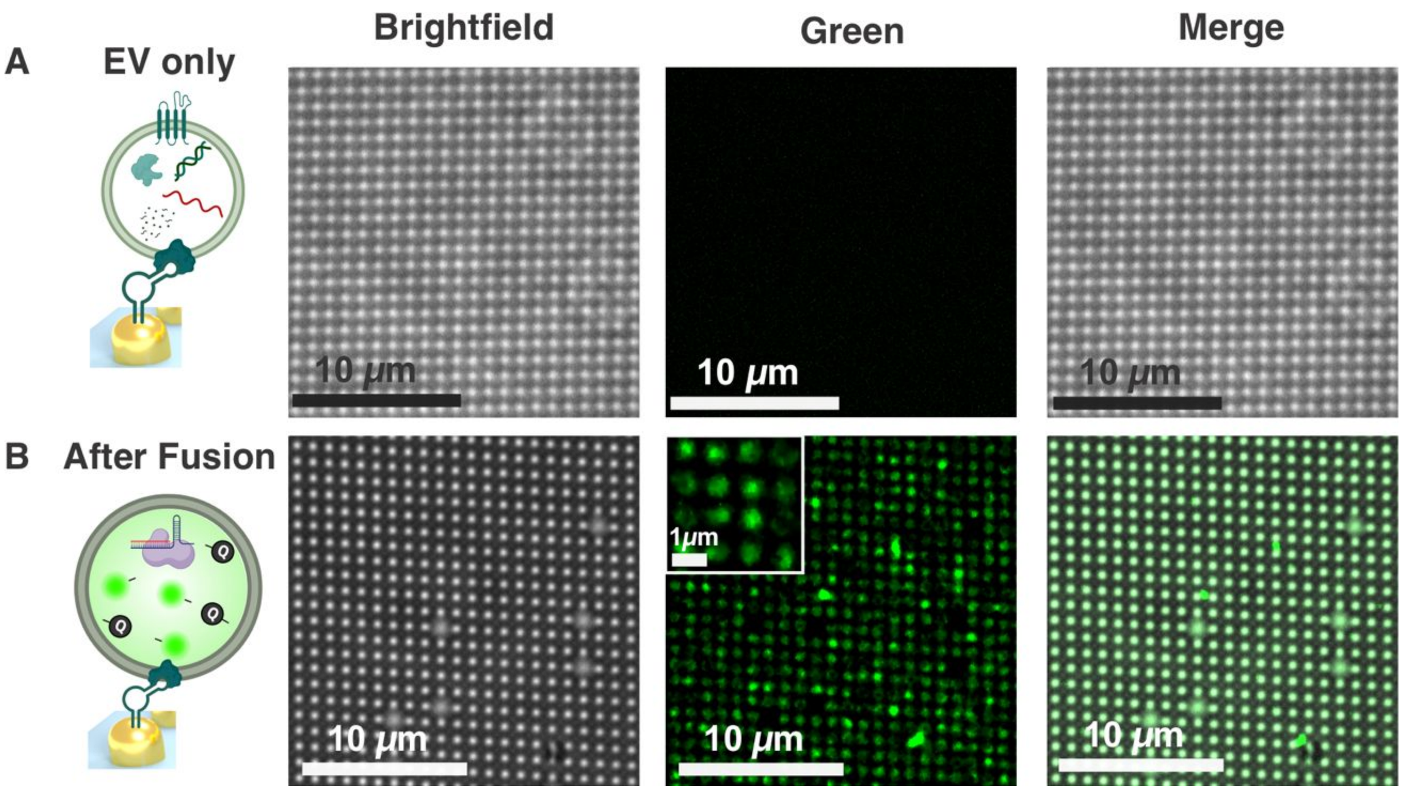

Figure S11. Confocal imaging of fluorescence detection using the fusion assay on a gold nanoarray. Representative confocal images of the EV-captured gold nanoarray before (A) and after (B) the fusion process. The fluorescence signal observed on the gold nanoarray after fusion (B) confirms the successful interaction between EVs and liposomes, enabling EV miRNA detection. Schematic created with BioRender.com.

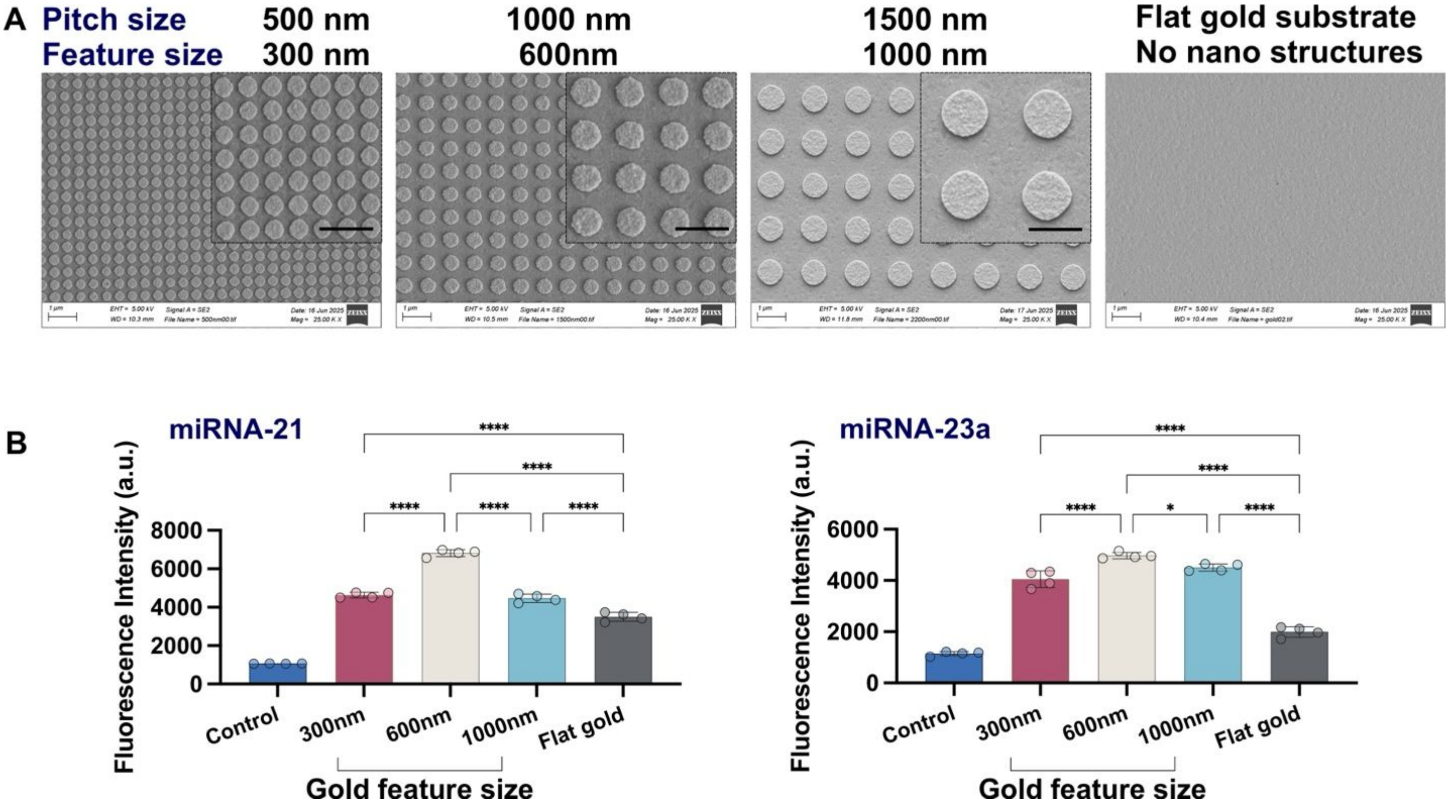

Figure S12. (A) SEM images of substrates with distinct gold feature sizes, including 300 nm, 600 nm, and 1000 nm nanostructures, as well as a flat gold substrate without nanostructures. Zoomed-in SEM images are shown in the insets. Scale bar: 1 μm. The feature size indicates the diameter of the gold nanostructures, while the pitch size refers to the distance between the centers of two neighboring gold nanostructures. (B) Fluorescence measurements of fusion assays performed on the different gold substrates. n=4, statistical analysis was performed using one-way ANOVA, * $p < 0.05$, **** $p < 0.0001$.

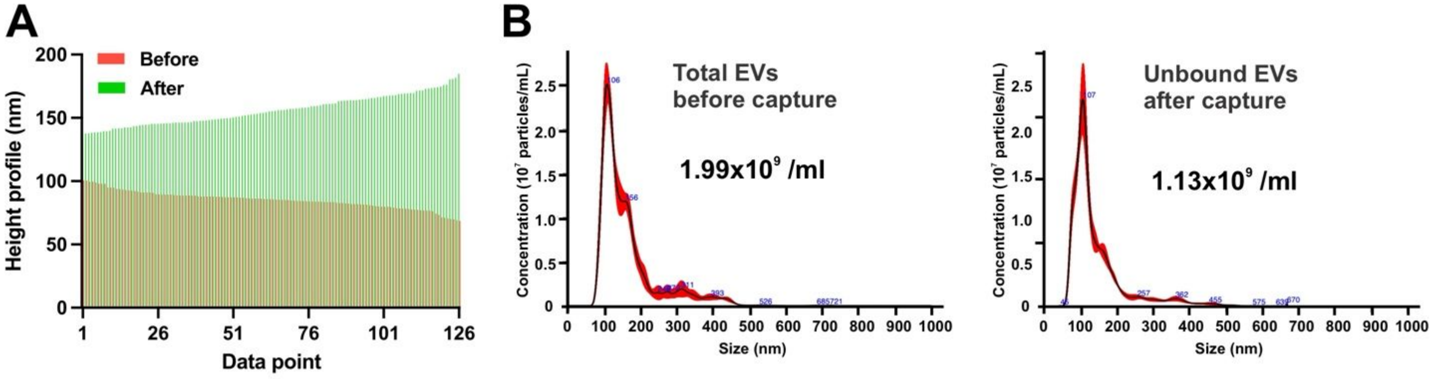

Figure S13. (A) Height profile measurement of the gold nanoarray before (red) and after (green) EV capture by AFM, with profiles collected from 126 points across the gold nanoarray surface. (B) NTA measurements of EV concentration before capture (total EVs) and after capture (unbound EVs), demonstrating efficient EV immobilization on the gold nanoarray.

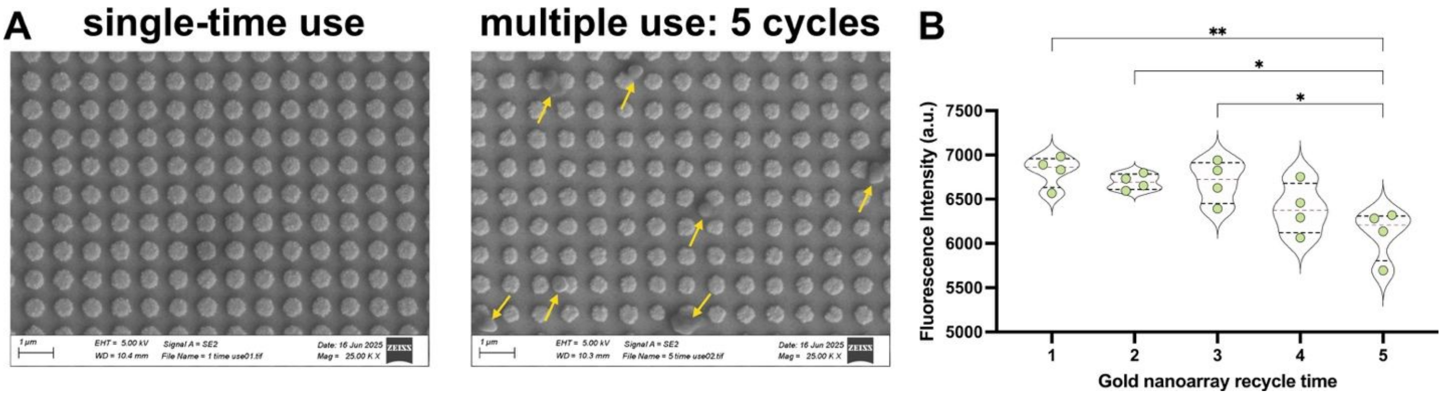

Figure S14. Reusability of gold nanoarray for EV detection. (A) SEM images of gold nanoarray after single use and five reuse cycles. Yellow arrows indicate the residues remained on gold nanoarray following 5 cycles of reuse. (B) Fluorescence performance of fusion assay on gold nanoarray at each repeated use cycle (n=4 per cycle, violin plots display individual data points, quartiles, and median values. Statistical analysis was performed using one-way ANOVA, * $p < 0.05$, ** $p < 0.01$).

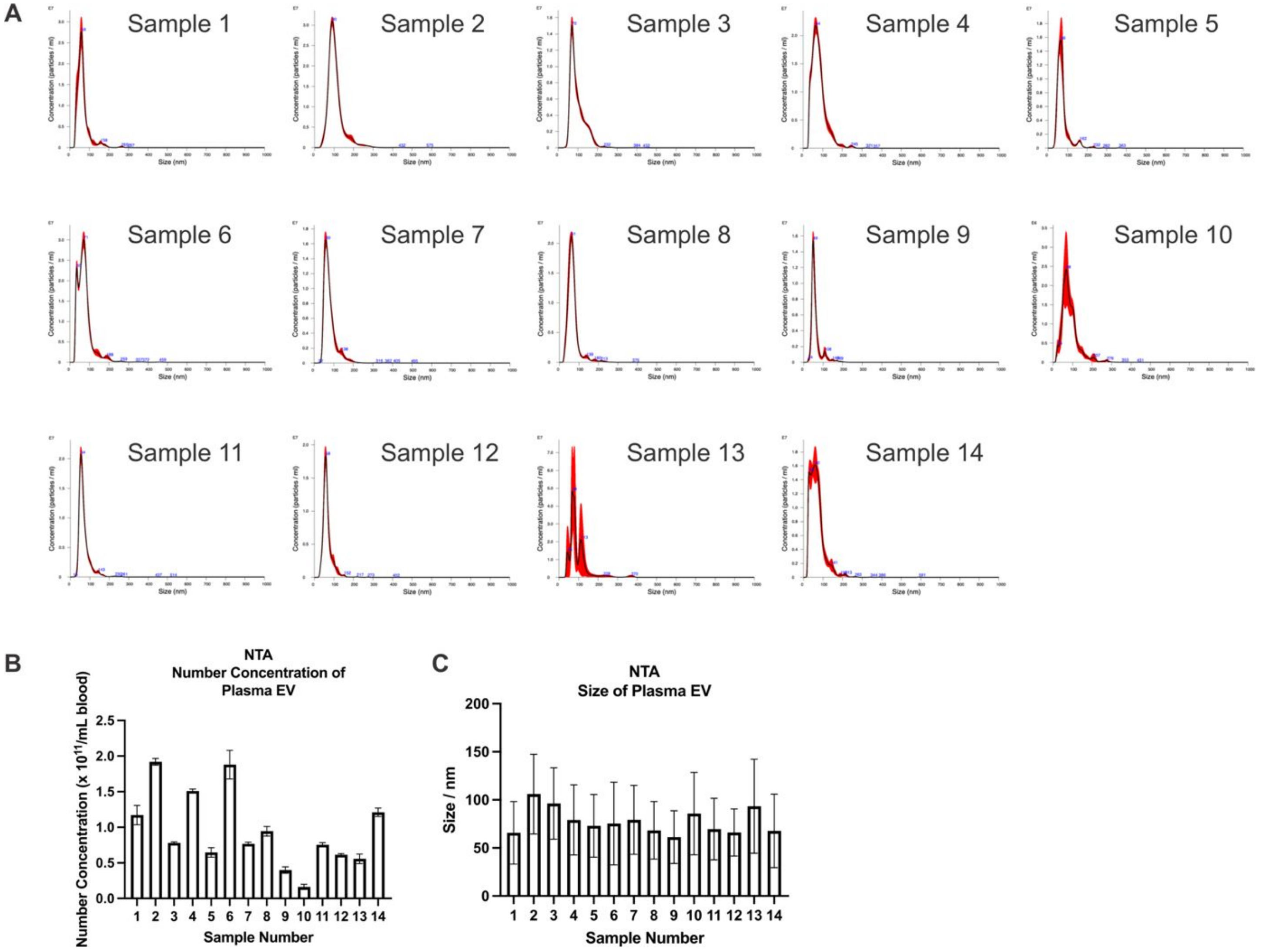

Figure S15. Characterization of human plasma EVs using NTA. (A) NTA size distribution analysis of human plasma-derived EVs diluted 100-fold in PBS. NTA analysis was performed with 5-time measurements. (B) Number concentrations of plasma EV concentrations. (C) Mean size of plasma EVs. Bar graph represents the mean ± SD (n=5).

Table S1: Lipid composition of synthesized liposomes

<table>
<tr><th rowspan="2">Lipid Composition (% in mol)</th><th colspan="2">FRET Assay</th><th rowspan="2">CRISPR/Cas13a-encapsulated Liposome</th></tr>
<tr><th>Fusogenic</th><th>Non-fusogenic</th></tr>
<tr><td>DMPC (non-fusogenic)</td><td></td><td>48%</td><td></td></tr>
<tr><td>DOPE (neutral, fusogenic)</td><td>48%</td><td></td><td>50%</td></tr>
<tr><td>DOTAP (positively charged)</td><td colspan="2">30%</td><td>30%</td></tr>
<tr><td>PEGlated DSPE</td><td colspan="2">5%</td><td>5%</td></tr>
<tr><td>Cholesterol</td><td colspan="2">15%</td><td>15%</td></tr>
<tr><td>TopFluor Cholesterol</td><td colspan="2">1%</td><td></td></tr>
<tr><td>Lissamine Rhod B DHPE</td><td colspan="2">1%</td><td></td></tr>
</table>

Table S2: Information on human subjects involved in this study.

Sample No.	Age	Gender	Histology	Stage	Pathology
**1**	54	Female	Colon Adenocarcinoma	T4N1	Colon CA
**2**	74	Female	Ovary with high-grade serous carcinoma<br>Rectal Adenocarcinoma (Dual Separate Current Dx)	Rectal = T1/2 N+	Colon CA
**3**	76	Male	Colon Adenocarcinoma	ypT3N2aM0	Colon CA
**4**	57	Male	Rectal Adenocarcinoma	T2N0	Colon CA
**5**	43	Female	Colon Adenocarcinoma	ypT3N2bM1	Colon CA
**6**	56	Female	Hypercellular Parathyroid (Hyperparathyroidism)	N/A	Non-CA
**7**	50	Female	Hypercellular Parathyroid (Hyperparathyroidism)	N/A	Non-CA
**8**	54	Female	Parathyroid Adenoma	N/A	Non-CA
**9**	29	Female	Adrenal Pheochromocytoma	N/A	Non-CA
**10**	29	Female	Thyroid Follicular Adenoma	N/A	Non-CA
**11**	43	Female	Complex Ovarian, Cervical, and Endometrial Cysts	N/A	Non-CA
**12**	33	Female	Thyroid with Graves Disease + Nontoxic Multinodular Goiter	N/A	Non-CA
**13**	38	Female	Hypercellular Parathyroid (Hyperparathyroidism)	N/A	Non-CA
**14**	77	Female	Kidney Sclerosis and Fibrosis from Chronic Pyelonephritis	N/A	Non-CA

Table S3: Summary of NTA analysis of Human plasma EVs.

Sample No.	Mean Size / nm	EV concentration (particles/ml blood)
**1**	65.8	$1.17\times10^{11} \pm 1.35\times10^{10}$
**2**	106	$1.92\times10^{11} \pm 4.71\times10^{9}$
**3**	96.2	$7.81\times10^{10} \pm 1.38\times10^{9}$
**4**	79.1	$1.51\times10^{11} \pm 2.62\times10^{9}$
**5**	73.0	$6.47\times10^{10} \pm 6.64\times10^{9}$
**6**	75.3	$1.88\times10^{11} \pm 2.01\times10^{9}$
**7**	79.2	$7.68\times10^{10} \pm 2.22\times10^{9}$
**8**	68.3	$9.44\times10^{10} \pm 6.77\times10^{9}$
**9**	61.3	$3.99\times10^{10} \pm 4.68\times10^{9}$
**10**	85.7	$1.63\times10^{10} \pm 3.64\times10^{9}$
**11**	69.6	$7.55\times10^{10} \pm 2.90\times10^{9}$
**12**	66.0	$6.14\times10^{10} \pm 1.69\times10^{9}$
**13**	93.4	$2.20\times10^{11} \pm 3.39\times10^{10}$
**14**	67.7	$1.21\times10^{11} \pm 5.88\times10^{9}$

Table S4: Sequence of oligomers used in this study.

Name	Sequence (5' to 3')
**miRNA 21**	UAGCUUAUCAGACUGAUGUUGA
**crRNA for miRNA 21**	GAUUUAGACUACCCCAAAAACGAAGGGGACUAAAACAACAUCAGUCUGAUAAGCUAUA
**miRNA 23a**	AUCACAUUGCCAGGGAUUUCC
**crRNA for miRNA 23a**	GAUUUAGACUACCCCAAAAACGAAGGGGACUAAAACGGAAAUCCCUGGCAAUGUGAUUA
**EpCAM aptamer**	/5ThioMC6-D/TTTTTTTTTCACTACAGAGGTTGCGTCTGTCCCACGTTGTCATGGGGGGTTGGCCTGT
**Poly U RNA reporter**	/56-FAM/UUUUU/3IABkFQ/

Table S5: Comparative summary of EV miRNAs detection.

Methods	Target miRNAs	Detection strategy	RNA extraction and amplification free	Assay duration	Specific detection of cancer EVs	LOD	EV sources	Ref
serum-stable membrane-fusion approach (SSMFA)	miRNA-21	Cas13a trans-cleavage	Yes	2h	Yes	1×10^7 particles mL^{-1}	2D cell culture: HepG2 human liver cancer cell<br>Animal model: Nude BALB/c mice blood	1
three-dimensional molecular beacon (ncMB)	miRNA-21	Molecule beacon	Yes	2h	No	1×10^9 particles mL^{-1}	2D cell culture: Malignant glioma cell: U251	2
liposome-mediated membrane fusion strategy (MFS)	miRNA-21	Cas13a trans-cleavage	Yes	1h	No	1.2×10^3 particles /mL	2D cell culture: Breast cancer cells: MCF-7<br>Clinical: human plasma	3
microfluidic cationic lipoplex nanoparticles (mCLN) assay	miRNA-21	Molecule beacon	Yes	10min	No	2.06×10^9 particles /ml	2D cell culture: A549 and BEAS-2B cells<br>Clinical samples: Human serum	4
3D microfluidic chip with vesicle fusion technology	miR-451a, miR-21, and miR-10b	Molecule beacon	Yes	2h	Yes	2.8×10^4 particles /ml	2D cell culture: pancreatic cancer cell line BxPC-1	5
High-throughput Nano-bio Chip Integrated System (HNCIB)	miRNA-21	Molecule beacon	Yes	6h	Yes	Not mention	2D cell culture: A549<br>Clinical sample: human plasma	6
DNA cage-based	miRNA-21,	DNA cage	Yes	2h	No	2.05 fM	2D cell culture: BT-474, MDA-	7

thermophoretic assay	miRNA-155						MB-231, and MCF-7<br>Clinical sample: human serum	
Aptamer-mediated selective fusion	miRNA-21	Molecule beacon	Yes	30min	Yes	0.14 μg mL^{-1} EVs	2D cell culture: A375<br>Clinical sample: human plasma	8
A virus-mimicking fusogenic vesicle (Vir-FV)	miRNA-21	Molecule beacon	Yes	2h	Yes	1.3 nM	2D cell culture: MCF-7<br>Clinical sample: human serum	9
Selective and amplification-free detection of EV miRNA by fusion assay on gold nanoarray	miRNA-21, miRNA23a	Cas13a trans-cleavage	Yes	1h	Yes	2.5×10^4 particles /ml	2D cell culture: human colorectal cancer cell SW480<br>3D vascularized tumor spheroid<br>Clinical sample: human plasma	This work

References cited in SI:

(1) Kong, H.; Chen, X.; Lee, W.; Xie, X.; Tao, Y.; Li, M. Dual-color fluorescence detection of tumor-derived extracellular vesicles using a specific and serum-stable membrane-fusion approach. *Biosens Bioelectron* **2025**, *278*, 117302.

(2) Mao, D.; Zheng, M.; Li, W.; Xu, Y.; Wang, C.; Qian, Q.; Li, S.; Chen, G.; Zhu, X.; Mi, X. Cubic DNA nanocage-based three-dimensional molecular beacon for accurate detection of exosomal miRNAs in confined spaces. *Biosens Bioelectron* **2022**, *204*, 114077.

(3) Zhang, J.; Guan, M.; Ma, C.; Liu, Y.; Lv, M.; Zhang, Z.; Gao, H.; Zhang, K. Highly Effective Detection of Exosomal miRNAs in Plasma Using Liposome-Mediated Transfection CRISPR/Cas13a. *ACS Sensors* **2023**, *8* (2), 565-575.

(4) Yang, Y.; Kannisto, E.; Patnaik, S. K.; Reid, M. E.; Li, L.; Wu, Y. Ultrafast Detection of Exosomal RNAs via Cationic Lipoplex Nanoparticles in a Micromixer Biochip for Cancer Diagnosis. *ACS Appl Nano Mater* **2021**, *4* (3), 2806-2819.

(5) Chen, X.; Jia, M.; Liu, L.; Qiu, X.; Zhang, H.; Yu, X.; Gu, W.; Qing, G.; Li, Q.; Hu, X.; et al. High-Fidelity Determination and Tracing of Small Extracellular Vesicle Cargoes. *Small* **2020**, *16* (40), e2002800.

(6) Zhou, J.; Wu, Z.; Hu, J.; Yang, D.; Chen, X.; Wang, Q.; Liu, J.; Dou, M.; Peng, W.; Wu, Y.; et al. High-throughput single-EV liquid biopsy: Rapid, simultaneous, and multiplexed detection of nucleic acids, proteins, and their combinations. *Science Advances* **2020**, *6* (47), eabc1204.

(7) Zhao, S.; Zhang, S.; Hu, H.; Cheng, Y.; Zou, K.; Song, J.; Deng, J.; Li, L.; Zhang, X. B.; Ke, G.; et al. Selective In Situ Analysis of Mature microRNAs in Extracellular Vesicles Using a DNA Cage-Based Thermophoretic Assay. *Angew Chem Int Ed Engl* **2023**, *62* (24), e202303121.

(8) Cui, L.; Peng, R. X.; Zeng, C. F.; Zhang, J. L.; Lu, Y. Z.; Zhu, L.; Huang, M. J.; Tian, Q. H.; Song, Y. L.; Yang, C. Y. A general strategy for detection of tumor-derived extracellular vesicle microRNAs using aptamer-mediated vesicle fusion. *Nano Today* **2022**, *46*, 101599.

(9) Gao, X.; Li, S.; Ding, F.; Fan, H.; Shi, L.; Zhu, L.; Li, J.; Feng, J.; Zhu, X.; Zhang, C. Rapid Detection of Exosomal MicroRNAs Using Virus-Mimicking Fusogenic Vesicles. *Angew Chem Int Ed Engl* **2019**, *58* (26), 8719-8723.
